# Understanding the implementation of a multidisciplinary intervention using a suite of prescribing safety indicators to improve medication safety in prison healthcare settings: a qualitative study

**DOI:** 10.1136/bmjopen-2024-086309

**Published:** 2025-03-05

**Authors:** Mark Jeffries, Aseel S M Abuzour, Darren Ashcroft, Tony Avery, Mark Langridge, Gayle Francis, Amber O’Brien, Tracy Millington, Richard Neil Keers

**Affiliations:** 1 Division of Pharmacy and Optometry, School of Health Sciences, The University of Manchester Faculty of Biology Medicine and Health, Manchester, UK; 2 Division of Population Health, Heatlh Services Research and Primary Care, School of Health Sciences, The University of Manchester Faculty of Biology Medicine and Health, Manchester, UK; 3 National Institute for Health and Care Research (NIHR) Greater Manchester Patient Safety Research Collaboration (GM PSRC), The University of Manchester, Manchester, UK; 4 Academic Unit for Ageing and Stroke Research, University of Leeds, Leeds, UK; 5 Primary Care, University of Nottingham, Nottingham, UK; 6 Health in Justice Practice Plus Group, Reading, UK; 7 His Majesty’s Prison Berwyn, Wrexham, UK; 8 Centre for Women’s Mental Health, University of Manchester, Manchester, UK; 9 Centre for Pharmacoepidemiology and Drug Safety Research, University of Manchester, Manchester, UK; 10 Suicide, Risk and Safety Research Unit, Greater Manchester Mental Health NHS Foundation Trust, Manchester, UK

**Keywords:** Safety, Quality in health care, Prisons, QUALITATIVE RESEARCH

## Abstract

**Abstract:**

**Objectives:**

Patients residing in prisons are a vulnerable group with more complex health needs and higher prevalence of inappropriate prescribing than the general population. Overcrowding in prisons, inadequate staffing levels, diversion of medication and substance misuse present challenges to prison healthcare. Interventions that use prescribing safety indicators are one way of helping to reduce the risk of harm by identifying patients at risk of potentially hazardous prescribing. This qualitative study aimed to understand the implementation and impact of a suite of seven prescribing safety indicators, specifically developed for use in prison settings, as part of a multi-disciplinary intervention.

**Design and setting:**

Semistructured interviews were conducted with a range of prison healthcare staff across 30 different prison sites in England. In addition, an online survey was made available to all healthcare staff in participating prisons. Data analysis of interview transcripts and free-text survey responses was conducted following a thematic approach and informed by normalisation process theory.

**Participants:**

Interviews were conducted with 9 prison healthcare staff and 40 completed the survey, with 18 staff providing free-text responses.

**Results:**

Three themes were interpreted from the data: bringing people together and establishing individual and collective roles that facilitated implementation of the intervention; developing new tasks, work processes and practices to make the intervention work in everyday practice; and seeing the benefits and value of the intervention and new work processes within the context of prison healthcare provision.

**Conclusions:**

New work processes and practices were instigated in order to implement the intervention, often fitting into existing medication safety practices, building on other prescribing work and creating learning across the team. While we found that prison staff reported challenges to implementation, similar interventions may be used for prescribing safety in prison settings.

**STRENGTHS AND LIMITATIONS OF THIS STUDY**

- Interview and survey participants were drawn from a diverse sample of prison staff involved in the delivery, or implementation, of the intervention across 30 participating prison sites.
- A particular strength of this study is the focus on normalisation process theory, which enabled a nuanced understanding of the ways in which the intervention was implemented.
- Those who volunteered to take part in interviews or the survey were most likely those using the intervention and thus potentially provided more positive responses.
- While a range of healthcare staff in different roles (general practitioners (GPs), pharmacists, advanced nurse practitioners) were interviewed, this included only one pharmacist and one GP, so there could have been greater breadth.
- The survey was completed in a limited fashion by the 40 respondents so only free-text comments could be analysed.

## Introduction

 Patients residing in prisons have greater mental and physical health needs than the general population.^1 2^ In the UK, increases in the proportion of prisoners aged over 50 will likely result in more prisoners suffering from age-related chronic diseases and polypharmacy.^3^ The prison population is more likely to have a history of substance misuse.^4^ In the UK, overcrowding in prisons, diversion of medication and inadequate staff levels present challenges to the quality of care provided.^3 5 6^ Patients residing in prisons have been seen to have a high prevalence of inappropriate prescribing rates and inappropriate polypharmacy.^7^ In two separate studies of prisons in England, Hassan *et al* found that age-adjusted prescribing of psychotropic medicines was up to 6 times higher for female prisoners and 5.5 times higher for male prisoners than in the general population.^8 9^ Elevated prescribing rates were found in the prison population in England, for the use of anticholinergics in patients aged over 65 (25.8%), prolonged use of hypnotics (46.3%) and the use of antiplatelets prescribed with non-steroidal anti-inflammatory drugs (NSAIDs) without gastric protection (12.5%–16.7%).^10^ In a cross-sectional analysis of 30 quality indicators in 13 prisons in England, McLintock *et al* found broad variations in the quality of primary care that were largely unexplained by available population characteristics.^11^

The increased use of information technology (IT) in healthcare presents an opportunity to enhance medication safety. Using prescribing safety indicators (PSIs), which can be searched using an electronic health record (EHR), is one way to proactively identify hazardous prescribing with the potential to reduce the risk of harm associated with medication.^12^ PSIs are statements describing ‘a pattern of prescribing that could be hazardous and may put patients at risk of harm’ and can be deployed in EHRs to proactively identify patients at risk of hazardous prescribing.^10 13 14^ PSIs have been incorporated into interventions that have successfully reduced potentially hazardous prescribing in primary care.^1214^,^18^

All prisons in England use the same EHR, which provides an opportunity to use PSIs across the prison estate. Our research team has previously developed and deployed PSIs to explore safer prescribing practices in prison settings. We identified the prevalence of hazardous prescribing in prison settings using PSIs deployed into two large, male, prison sites in England and Wales and explored the implementation and use of PSIs in practice.^10^ 13 PSIs were successfully deployed within the prison EHR. Prison healthcare staff were supportive of the use of PSIs to improve prescribing practices but emphasised the need for a designated member of staff to conduct the PSI search. Healthcare staff expressed a preference for responding to PSI data collaboratively with other prison healthcare staff.^10^ A qualitative study by the research team has also characterised the processes and challenges to safe prescribing in prisons.^6^ Prescribing in prisons presented unique challenges, not found in primary or secondary healthcare settings, impacted by the complex health needs of prisoners, patient behaviour, trading of medicines, transient populations, staff retention and problematic IT.^6^

This study aimed to understand the implementation of a suite of seven PSIs embedded within a multidisciplinary intervention to improve medication safety in prison healthcare settings.

## Methods

### Study design

This was a qualitative study using semistructured interviews and free-text survey responses.

### The intervention

The intervention was in prison sites where a national independent sector prison healthcare provider delivered healthcare and was conducting PSI searches within its prison estate, using an indicator suite made up of approximately 50 indicators. Building on our previous work^6 10^ and earlier research implementing PSIs into primary care, we developed an intervention which aimed to reduce the number of patients affected by seven of our prison-specific PSIs in prisons across England. Our seven PSIs comprised risks associated with commonly prescribed medicine combinations and important monitoring requirements, particularly concerning cardiovascular and mental health (box 1).

Box 1 **Prescribing safety indicators (PSI) developed for prison settings^10^**

**PSI**

- Coprescribed opiates with methadone/buprenorphine.
- Coprescribed opiates with pregabalin/gabapentin.
- Prescribed benzodiazepine, Z-drug or sedating antihistamine for more than 1 month.
- Prescribed selective serotonin/norepinephrine reuptake inhibitors (SSRI/SNRIs) with non-steroidal anti-inflammatory drugs (NSAID) or aspirin with no gastrointestinal protection.
- Antiplatelet prescribed to a patient concomitantly with an NSAID without gastroprotection.
- A medication with medium/high anticholinergic activity prescribed to a patient aged ≥65 years.
- Antipsychotic prescribed for at least 12 months without monitoring blood glucose, weight or lipid profile within the previous year.

**Figure 1:**
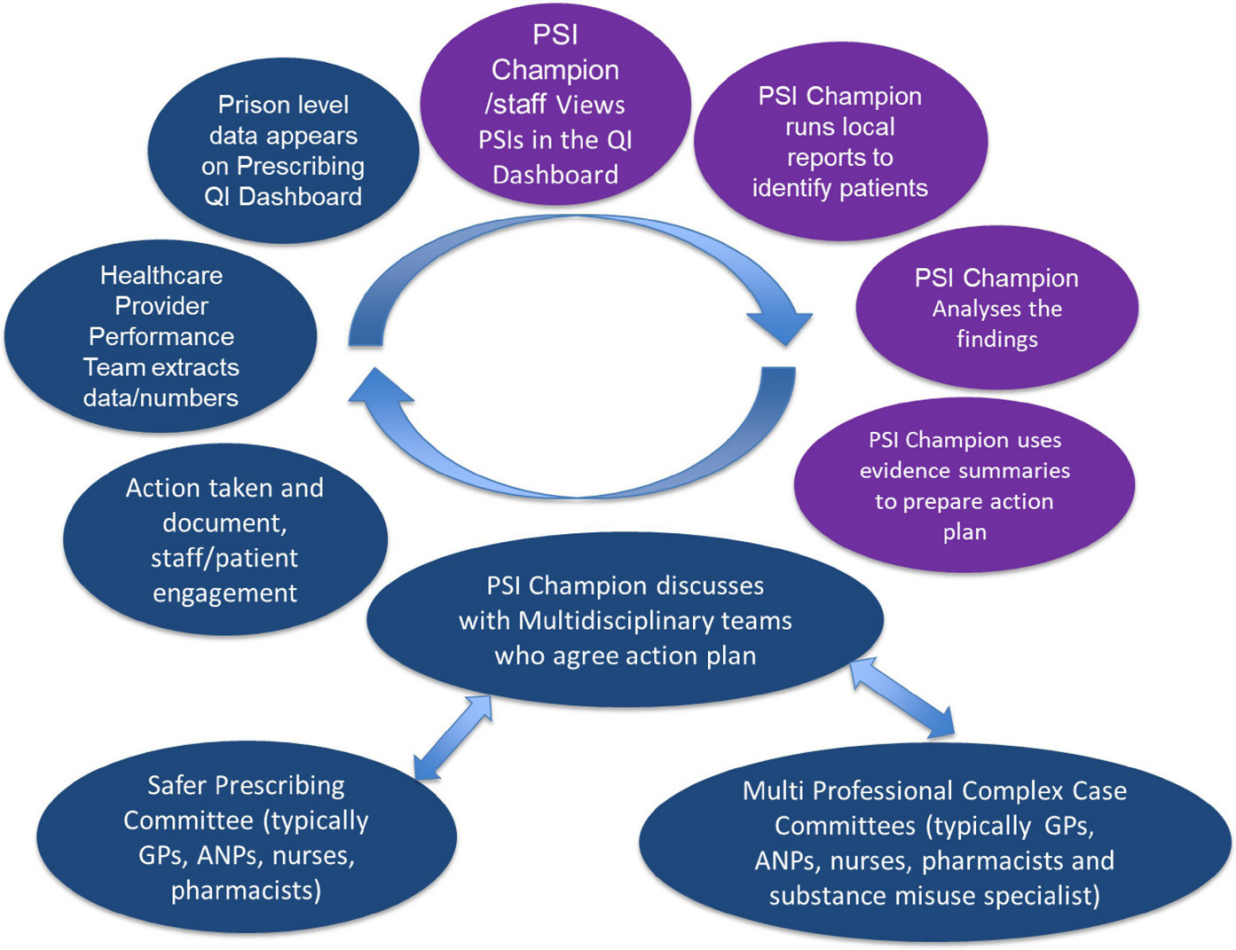
The intervention. ANP, advance nurse practitioner; GP, general practitioner; PSI, prescribing safety indicator.

The intervention aimed to identify patients at risk of hazardous prescribing from the EHR system used in prisons, review the medication of these patients through a multidisciplinary team (MDT) and develop an action plan to reduce the number of patients at risk. The MDT at each prison was led by a designated ‘PSI champion’, a trained healthcare member of staff with a consistent role at the prison. Training of the PSI champions was led by the research team at the University of Manchester and adapted from two large studies that the research team had previously completed.^12 14^ The PSI champion ran searches locally in their prison to identify patients exposed to potentially hazardous prescribing or inadequate monitoring as defined by the seven PSIs. This provided an opportunity to prepare actionable feedback at an individual patient level and the preparation of a draft action plan to be discussed with the wider MDT.^14^ The intervention involved a cyclical process in that it meant once actions were completed, further searches could be undertaken (see figure 1).

### Recruitment to the intervention

All prisons of the national independent sector prison healthcare provider received the seven indicators and were encouraged to use them. Of those, 30 prison sites were included in the evaluation after excluding institutions where prisoners were not adults, or whose sentences were of such a short duration that the intervention was considered unlikely to be effective (security category B remand prisons, youth offender institutions and immigration sites).

### Sampling and recruitment of participants

Interview and survey participants were drawn from a purposive (interviews) and convenience (survey) sample of prison staff involved in the delivery or implementation of the intervention at participating prisons. This included prescribers (general practitioners (GPs), non-medical prescribers, psychiatrists), pharmacy team members, other prison healthcare professionals (eg, substance misuse staff). Participants were informed about the study through email distributions from the prison healthcare provider, study adverts placed in clinical areas of participating prisons and promotion by the research team at the PSI Champion forum and local and regional prison meetings. Participants, interested in taking part in an interview, contacted the research team directly by email and were then emailed participant information and a consent form. Consented participants were then contacted to arrange the interview.

### Data collection

The interview schedule and survey questions (onlinesupplemental files 1  2) were informed by normalisation process theory (NPT)^19^,^22^ to understand how participants made sense of the intervention, the ways it was operationalised in prison sites, the collaborative and interactional work involved in the adoption of the intervention and how it was appraised. Interviews were conducted by MJ (an experienced qualitative researcher) using an online digital platform (Microsoft Teams). Interviews were recorded, with participant consent and transcribed verbatim. The online survey was piloted within the project team, including prison and mental health pharmacists, with minor amendments made to improve clarity before launch. The survey was also based on the established and validated NPT survey tool available online.^23^ The survey comprised a mixture of Likert scale and free-text responses and was made available to all healthcare staff at participating prison sites staff via a URL link circulated through the platform Qualtrics from June 2022 and closed in August 2022. Both the interviews and survey were anonymous, but job titles were recorded along with the prison site where the participant was based.

### Data analysis

#### Framework

NPT is concerned with explaining the work people do and focuses on the social processes of intervention implementation and how they become part of everyday practice as new behaviours are adopted into routine work.^19^,^22^ NPT is built on four constructs: coherence—the way the intervention is understood, cognitive participation; the way people work together to put the new intervention into practice; collective action—the ways people work in operationalising the intervention; and reflexive monitoring—how people evaluate the new practice and work to sustain it. Each construct is built on four components which give further nuanced explanation of the ways interventions might be accommodated into everyday working practices.^19 21^ These constructs and components are discussed further in table 1.^19^,^22^ NPT has been previously used to understand a number of healthcare interventions, including those involving prescribing safety and PSIs.^1624^,^26^

**Table 1:** Normalisation process theory (NPT): constructs and components^21^

NPT construct	Component	Explanation
CoherenceSense-making work: understanding and conceptualisation of interventions and their work.	Differentiation	What people do to understand how a set of practices and their objects are different from each other. What they do to organise the differences.
	Communal specification	People working together to build a shared understanding of the aims, objectives and expected benefits of a set of practice. How a team works out how to integrate an innovation into their healthcare setting.
	Individual specification	Individuals’ understanding of their specific tasks and responsibilities around a set of practices.
	Internalisation	Work to understand the value, benefits and importance of a set of practices. The *w*ork people do to attribute worth to a new way of working.
Cognitive participationRelational work that people do to build and sustain a community of practice around a new technology or complex intervention: notions of legitimation and buy-in, both in terms of the individuals involved and involving others.	Initiation	The work people do to drive forward the new or modified practice. Setting things up and working with others to make things happen.
	Enrolment	How participants organise and reorganise themselves and others in order to collectively contribute to the work involved in new practices. This is complex work that may involve rethinking individual and group relationships between people and things.
	Legitimation	The work ensuring that other participants believe it is right for them to be involved, and that they can make a valid contribution to it.
	Activation	The work of keeping the new practices in view and connecting them with the people who need to be doing them. Collectively defining the actions and procedures needed to sustain a practice and to stay involved.
Collective actionOperational work that people do to enact a set of practices: organisational resources, training, division of labour, confidence and expertise as well as the workability of the intervention in clinical interaction	Interactional workability	The interactional work that people do with each other, with artefacts and with other elements of a set of practices, when they seek to operationalise them in everyday settings. The impact the new practice has on interactions with each other and/or service users.
	Relational integration	The knowledge work that people do to build accountability and maintain confidence in a set of practices and in each other as they use them. The impact the innovation has on relationships between different groups of professionals for example, trust, accountability and responsibility.
	Skill set workability	The allocation work that underpins the division of labour that is built up around a set of practices as they are operationalised in the real world. Who gets to do/did what and how the tasks relate to their existing skill sets.
	Contextual integration	The resource work—managing a set of practices through the allocation of different kinds of resources and the execution of protocols, policies and procedures. Fit between the new practice and overall organisational context, including organisational goals, morale, leadership and distribution of resources (eg, funding, policy and priorities).
Reflexive monitoringAppraising and monitoring implementation work. The appraisal work that people do to assess and understand the ways that a new set of practices affect them and others around them.	Systematisation	The work of collecting information in a variety of ways to determine how effective and useful the new practice is for them and for others.
	Communal appraisal	Participants work together—sometimes in formal collaboratives, sometimes in informal groups to evaluate the worth of a set of practices.
	Individual appraisal	Individuals appraising the new practice in relation to their own work; the impact it has on their tasks. Actions through which individuals express their personal relationship with the innovation.
	Reconfiguration	The appraisal work by individuals or groups which may lead to attempts to redefine procedures or modify practices—and even to change the shape of the innovation itself.

#### Thematic analysis

The survey was only fully completed by 18 staff members. It was, therefore, decided to analyse only the free-text comments from the survey thematically alongside the interview data.

MJ led on the data analysis, which was conducted concurrently and iteratively alongside data collection. Data collection continued until saturation was reached and no new themes could be interpreted from further interviews. Analysis initially followed a thematic approach informed by Braun and Clark^27^ and subsequently a template approach.^28^ The first six interview transcripts were first read and re-read and then inductively coded by MJ using QSR NVivo V.12 to organise the data. This allowed for the identification of patterns, groups of codes and potential themes. These were then further discussed by MJ and RNK. The analysis then moved to a template approach with the codes then organised and incorporated into a table to create a new coding framework. This framework was then applied to the full data set of interviews and survey free text comments. In a confirmation exercise, author ASMA independently read all transcripts, the survey free-text comments, the completed coding framework and identified themes. These were then further discussed between RNK, ASMA and MJ in order to refine, interpret and then confirm the final group of themes presented here.

### Patient and public involvement

The project team included a lived experience representative (TM). They contributed to the development of the project plan and attended project meetings.

## Results

A range of prison healthcare staff (N=9; 6=advance nurse practitioners (ANP), 1=pharmacist, 1=GP, 1=clinical lead) took part in eight interviews. Interviews ranged from 28 min to 44 min in length and took place in May and June 2022 (see table 2). The survey was completed by 40 prison healthcare staff, with free-text responses collected from 18 respondents from participating prisons (see table 2). Likert scale data were poorly completed by respondents and was therefore excluded from the analysis. At 22 of the 30 sites involved in the intervention, a PSI champion was in place and work had commenced responding to the PSI reports during the implementation period (May to August 2022). At the other eight sites, inactivity was explained by, among other reasons, a lack of staffing, no PSI champion to lead the intervention or high workload.

**Table 2:** Interview and survey participants

Participant ID	Participant role	Prison category*	Individual or joint interview/survey response
PSIP1_ANP1	Advanced nurse practitioner	C†	Joint
PSIP2_ANP2	Advanced nurse practitioner	C	
PSIP3_Clinical Lead1	Clinical lead	Female	Individual
PSIP5_ANP3	Advanced nurse practitioner	A	Individual
PSIP6_ANP4	Advanced nurse practitioner	A	Individual
PSIP7_Pharmacist1	Pharmacist	C	Individual
PSIP8_ANP5	Advanced nurse practitioner	A	Individual
PSIP9_GP1	General practitioner	C	Individual
PSIP10_ANP6	Advanced nurse practitioner	Female	Individual
S1	Regional mental health lead	n/a	Survey
S2	Bank registered general nurse	Not known	Survey
S3	Healthcare assistant	B, C	Survey
S4	Nurse	C	Survey
S5	General practitioner	C	Survey
S6	Head of healthcare and non-medical prescriber	B	Survey
S7	Mental health nurse	C	Survey
S8	Nurse	Unknown	Survey
S9	Healthcare assistant	C	Survey
S10	Lead pharmacist	B	Survey
S11	Projects manager	B, C	Survey
S12	Pharmacy technician	C	Survey
S13	Lead pharmacy technician	B	Survey
S14	Advanced nurse practitioner	C	Survey
S15	Pharmacist	C	Survey
S16	Pharmacy technician	C	Survey
S17	Pharmacy technician	C	Survey
S18	Pharmacy manager	B	Survey
S19	General practitioner	B	Survey
S20	Clinical lead	C	Survey
S21	Clinical lead	D	Survey
S22	Registered nurse	D	Survey
S23	Clinical lead for substance misuse	B, C, D	Survey
S24	Pharmacy technician	D	Survey
S25	Clinical lead	D	Survey
S26	Mental health nurse	B	Survey
S27	Head of healthcare	B	Survey
S28	Pharmacy operational manager	C, D	Survey
S29	Primary care clinical lead	Female	Survey
S30	Mental health administrator	C	Survey
S31	Pharmacy technician	D	Survey
S32	Mental health clinical lead	C	Survey
S33	Pharmacy technician	C	Survey
S34	Patient safety and clinical quality lead	B and C	Survey
S35	Long-term conditions nurse	B	Survey
S36	Clinical lead for substance misuse	B, C	Survey
S37	Regional primary care lead	n/a	Survey
S38	No role given	n/a	Survey
S39	No role given	n/a	Survey
S40	Senior primary care nurse, bank nurse	B, C, D	Survey

* Prisons in England and Wales are categorised depending uponon security level.

† Category A prisons are high-security facilities. They house male prisoners who, if they were to escape, pose the most threat to the public, the police or national security. Category B prisons are either local or training facilities. Category C prisons are training and resettlement facilities. Category D prisons are open facilities with minimal security and allow eligible prisoners to spend most of their day away from the prison on licence to carry out work, education or for other resettlement purposes. Female prisoners are housed separately in open or closed conditions.^43^

ANPadvance nurse practitionern/anot available

NPT helped to inform the interpretation from the data of 11 subthemes grouped into 3 main themes, as summarised below. Further details of these and additional exemplar extracts from the interviews and survey are given in online supplemental file 3. These themes were interrelated to the components and constructs of NPT.

- Bringing people together and establishing individual and collective roles that facilitated implementation of the intervention.
- Developing new tasks, work processes and practices to make the intervention work in everyday practice.
- Seeing the benefits and value of the intervention and new work processes within the context of prison healthcare provision.

### Bringing people together and establishing individual and collective roles that facilitated implementation of the intervention

People coming together in teams facilitated the implementation of the intervention. ‘Buy in’ and a willingness to engage were perceived as important since the intervention required a level of organisation and collaboration across different professional groups, agreement over actions, such as changing or stopping medications and who would undertake those actions.

…meeting regularly as a team of prescribers and professionals has gone really well.[…] we’ve had such buy-in from the GP, I think that’s been brilliant for us. […] so regular meetings, buy-in from the team and the gatekeeping from pharmacy, they would be our three main positive aspects. Clinical Lead1

It was perceived that an effective way of working to deliver the intervention was to have MDTs. Participation in groups and meetings promoted discussion of medication safety issues identified by the prescribing indicator searches, and the intervention *“required the willingness of the entire team to be involved”ANP3.* Similarly communication was seen as important, “*… so we have that consistency of approach”* and *“communicate what everyone else is doing” ANP3.* Teamwork could involve managerial and clinical supervision with individual prescribers, broader medicines management meetings or a mix of different professionals and professional groups. This helped in sharing full information, ensuring understanding across the team and that potential interventions for patients affected by prescribing indicators were not missed. This provided both a stimulus and impetus to implementation and a process of checking that the intervention was on track.

In medicines management meetings we would highlight the number of incidents and trends. I would also share the full information with the lead GP (to identify each individual prescriber) after the meeting, so that these issues could be discussed in managerial/clinical supervisions with the individual prescribers. Patient Safety & Clinical Quality Lead (Survey free text)

Within the wider teams having an individual to drive the intervention was important. This was often the designated ‘PSI Champion’. Where one person was leading on the intervention, it was felt that there needed to be input from the team because of the perceived complexity of the intervention and *“the long term use of (some of these) medicines”ANP3.* Sometimes the person leading the implementation of the intervention was the only person involved. This could lead to workload issues and working in isolation which made “*keep[ing] things going”* with the intervention difficult, as described by this GP:

I need to run a [prescribing safety indicator] report, and then I need to look at these patients, and I need to decide whether or not I should prescribe differently, and then I need to review the patient as an addition, potentially, and I need to change the prescribing. I couldn’t see anybody within the team that we have now, that I could really pass much of that workload onto. GP1

This isolation in leadership was reflected in the survey response of a pharmacy technician “(The) pharmacist oversees (the) PSI (intervention) otherwise no-one seems to be aware of it or what it involves” Pharmacy Technician (Survey free text).

There were perceptions that leadership of intervention implementation needed to come from ‘somebody that’s in a clinical job role’ since it was thought to ‘require(s) a lot of using your clinical knowledge, identifying high risk patients’ and prepare actions. (Pharmacist1)

For some, this leadership, it was thought, should come from a clinical pharmacist. That some sites had no full-time pharmacist was used as an explanation for why progress on implementation of the intervention had been slow; if there was *‘plenty of pharmacy time’* the intervention would *‘fit beautifully’* and that tasks such as checking the reports, reviewing notes, discussing with the patient would be straightforward to conduct. Other sites felt that capacity to *‘fully embed’* the intervention was limited since the prison had been *‘without a lead Pharmacist for a prolonged period’ (PSI Head of Healthcare (Survey free text).*

### Developing new tasks, work processes and practices to make the intervention work in everyday practice

For the intervention to work successfully and to be normalised into everyday practice, new tasks, processes and work practices were adopted. This could involve new solutions, often instigated by ‘PSI Champions’, such as creating personalised and localised systems in order to track indicator-related actions and keeping records of patients identified by the PSIs by, for instance, using spreadsheets.

…so every month I will refresh it, and I keep a record …because what I do is I’ll jot down what it was last month, so say, for example, last month I did it on 20th, I’ll jot it down and I’ll keep a track of whether our figures are going up or down. Pharmacist1

Work processes were collective, and systems were introduced to allow for the sharing of information from the PSI reports. These systems could be based around regularly running reports or collaborative working. For one pharmacist, this involved sending the indicator report each month to a Substance Misuse Service Nurse who could target “*these patients (highlighted in the PSI report) in a safer prescribing clinic”* and book them in to the clinic. In this way, *“the report’s identified what patients we need to target”. Pharmacist1*

Creating new work practices, within already busy work schedules, meant there was a time factor in implementing the intervention. While, for this ANP, the intervention was not seen to involve a lot of work and could be achieved *‘once you actually got on top of it’,* it did require assigning specific time to complete tasks.

Like I say I work from home on a Friday for an hour and a half, that’s my hours, so that’s when I do the report so it only takes me about an hour and a half to do the reports and action anything and things like that. ANP1

It was also seen that flexibility was required because of other demands on time. In this way, the intervention was fitted alongside existing work demands and time constraints but required regular input to avoid losing momentum *‘and a bit of continuity’ (ANP6)*.

[…] we also have a lot of task things coming though of prescriptions, requests for certain things, so we have to be quite flexible … even if I set an hour aside to do something, there might be an urgent patient to see, urgent tasks, and on Mondays I also have to go to segregation unit. […] (so) trying to fit this in, because the moment you lose a bit of continuity, it’s harder to get back into it. ANP6

Other participants felt that workload demands made the intervention difficult to fit in since it was seen as an additional thing to do and that they were already somewhat isolated ‘*without any other prescribers or a pharmacist, or anyone else on board as such’ GP1*

So while time was important, it was related to workload. Having support could ensure that the intervention could continue since other staff, other than the lead or PSI Champion, were available to take over if required.

And (name) knows how to run the [PSI] report so if for any reason I’m off (name) will be able to run the report for me and ensure that it’s all updated. […] If for some reason I wasn’t here, (name) would be able to still complete and run the reports. ANP1

The intervention was reported to fit into existing medication safety interventions, existing work structures, such as ‘meetings that already exist’ at some participating prisons, to avoid creating ‘massive amounts of workload’ (Pharmacist1). For some, the intervention was new and different, but others had previously worked with PSIs, and that allowed them to respond to the intervention more impactfully. The PSI intervention was described to complement and build on other safer prescribing work but provide a clearer identification of those patients who required a review.

…our dashboard will say, you’ve got 70 patients on Amitriptyline or you’ve got 120 patients on Methadone and 30 on Pregabalin, but it never gives you a real breakdown, because what these PSI reports do, it gives us a breakdown of the patients […] that need to be having these reviews… 111

The intervention involved allocating tasks to those with the most appropriate skill set to undertake them. This ANP highlighted how one member of staff was able to draw on existing knowledge of patients so was well placed to work on making changes to medications and undertaking monitoring tests of patients highlighted by the PSIs.

And the mental health team, and we've got a pharmacy tech(nician) up there […] So it was one of our biggest lists, the mental health and the bloods (tests), so rather than me have to do it, (they) know (their) patients better than what I do, so (they) did the original scan of all the notes to make sure they had the bloods, lipids and weights taken. ANP1

### Seeing the benefits and value of the intervention within the context of prison healthcare provision

Participants found the intervention of value and that there were reported direct benefits to patients. Prisons had specific challenges that impacted on how the intervention was delivered. The intervention impacted on communities of practice by contributing to wider medication safety work, and individual and group learning. The intervention was considered *‘quite non-judgemental’* and was not perceived as highlighting individual performance to *‘say, you’ve done bad prescribing on this’* but was considered *‘a good systems check’. (GP1)*. The intervention was seen to extend work, to look more broadly at patient safety and ‘*making us more aware of some of the issues that previously might have just gone and been ignored’ (ANP4)* and, as this ANP reported, helped people become safer prescribers.

So I think with the PSI (patient safety indicator) reporting, especially with bloods and things it makes us more aware […], it allows us to ensure safety for the patients. And it’s not about, stopping med(icine)s as to whether they are right or wrong, it is exactly down to that, it’s patient safety. So I think more than anything, this has just helped us to be safer prescribers.ANP1

The intervention was seen as benefiting and facilitating learning across, and within, teams, as this pharmacist reported, specifically in the prescribing of NSAIDs.

…that’s something that I took to my pharmacy meeting and shared some of the learning with my pharmacy team, was it identified patients that were prescribed NSAIDs and then were given NSAIDs as part of the minor ailments protocol. […]that was a lot of learning for…some invaluable learning for my (pharmacy) technicians as well, from the (PSI) report…. Pharmacist1

Participants reflected and judged the effectiveness of the intervention within the context of challenges that were specific to prison settings. Patients were characterised as having ‘*very traumatic backgrounds’* and being a *‘very vulnerable group’* who prior to their being in prison had ‘*not had access to healthcare’ (ANP6).* Patient engagement was seen as really important and that it was thought that *‘patients need to be more involved from the very start, so they’re aware of the risks of taking these drugs and they’re not just prescribed and put on a repeat (Pharmacist1).* However, participants reflected that the characteristics of the prison population made engagement difficult, particularly when it came to changing prisoners’ medications. This ANP reported how they used a multidisciplinary approach to aid their engagement:

We’ve had a few malicious threats about it (changing prisoners medications), so we’re aware of security issues and safety issues, […] there’s a couple of complex patients that are still challenging some of our decisions through the [meeting].ANP3

Safety was a consideration when engaging with high-risk offenders but had to be balanced with issues of confidentiality with finding ways to talk to patients as this same ANP discussed;

…to see someone face to face is better. And we’ve started doing that more and more, or even just speaking at a cell door, is often the way that I will do it now, where, yeah, there’s maybe a little bit of confidentiality, it’s less ideal, ‘cause you’re on the wings, but it means my safety’s guaranteed.ANP3

Alongside these challenges, the intervention was seen as having a positive impact on the lives of patients. The intervention was seen as making a positive difference in that healthcare staff were now *‘able to find those patients and actually improve their treatment’ (ANP5*). In this example, from a pharmacist reviewing a patient after investigating one of the PSI reports, changes in the patient’s medicine were made to avoid distressing weight gain.

…when we pull the PSO4 (see box 1) report, the nurse came to have a word with me saying that she’s actually gone in to do some weights for (a patient) and realised his weights per month increased because Mirtazapine, one of the side-effects is weight gain […] it was quite distressing for him, the weight gain. So we swapped him onto a different alternative. Pharmacist1

Benefits of the intervention were similarly found in that it presented opportunities ‘*to identify patients who would benefit from a meds review’*, and to ensure *‘that patients are receiving effective care, safe care’* and that *‘we can actually address those health needs’ ANP6*

Participants reflected on the challenges that had arisen in implementing the intervention. This included fatigue and discouragement within the team. It was suggested that since ‘*it is hard to maintain, when you’ve got a client group that are demanding certain medications’(ANP3),* it was important to have ‘*strength within the team, and resilience within the team, to keep going through that’ (ANP3).*

## Discussion

This study aimed to understand the implementation and impact of a suite of seven PSIs with accompanying medication safety improvement intervention in prison healthcare settings. The successful implementation of the intervention was seen as dependent on stakeholders working together in MDTs and on the development of new work processes and practices. The intervention was understood within the organisational contexts of prison settings. This builds on our previous work in prison settings by further understanding how the complexity of prison settings might impact on how medication safety work is both undertaken and achieved.^6 10^ As with previous work, we also found that staffing and staff issues, particularly staff safety, impacted the implementation of the PSI intervention and wider prescribing safety practices. Staffing and workload issues could mean intervention activities were not completed because of excessive workloads and MDT meetings not being held. At an individual level, staff safety could impact on discussing changes of medications with patients. This relates to how avoidable harms in prisons have been seen to be impacted by workforce or staffing issues.^29^

PSIs, like other IT interventions do not, however, operate in isolation from the social and organisational contexts in which they are implemented, and the utilisation of IT has therefore been understood as a social practice.^30^,^35^ The ways in which this intervention was embedded within organisational contexts are therefore an important finding here. As reported by some respondents, the PSI reports afforded technological solutions which could then be acted on. Furthermore, individuals created their own solutions in terms of new work practices and work processes, which could be shared with others in communities of practice. Such reciprocal and recursive movement between the technology—its affordances—and the work and organisational practices has been previously observed in the context of medication safety and technological interventions in primary care.^30 32 36 37^

We found that the intervention was particularly reliant on an MDT and that where that team was in place, the intervention was more likely to prove beneficial and impactful. Similarly, leadership was particularly important, and having a key individual to lead the intervention, without whom the implementation could lose momentum. Such organisational structures were the contextual background in which the intervention was being implemented. Leadership, collaboration or team structures have been seen to be important for medication safety interventions previously in healthcare settings, including in repeat prescribing^37^ and other PSI interventions.^16 18^ In the present study, leadership was considered important and particularly from someone with a clinical background. This is interesting in that technology interventions have been previously seen to impact on professional roles and reinforce differences between professional groups.^38^ It could be potentially limiting to medication safety interventions, and those using PSIs, if they were solely led by, and seen as the responsibility of, prescribers, pharmacists or GPs, as found previously^16^ and as described in the present study, by those with a clinical background. This is because wider healthcare staff may not have had significant input in intervention delivery.

### Implications for practice, policy and further research

Our study raises a number of implications for policy practice and further research and supports the wider exploration and use of prescribing safety or quality indicators to assess, or as interventions for improvement, in UK prison settings^11^ (see box 2). Overprescribing in healthcare has been highlighted as a serious problem and a potential cause of patient harm and inappropriate polypharmacy.^39^ Suggestions for improving this include medicine optimisation via structured medication reviews and deprescribing.^39^ Since our intervention involves searches and review, it has the capacity to be of use in this context. The unique prison environment, where one EHR is used ubiquitously and where multiprofessional teams work in one place, allows great potential for opportunity for this type of intervention. Similarly, our intervention is a system-based approach, and it has been suggested that digital tools and system approaches would be beneficial at reducing overprescribing.^39^

Box 2 **Key considerations for implementing similar prescribing safety indicator (PSI) interventions**

**Key considerations for implementing similar PSI interventions**

- Use a multidisciplinary team.
- Provide key leadership (either group or individual).
- Ensure regular meetings occur involving multidisciplinary team to review patients and plan actions.
- Call on the most appropriately skilled to allocate tasks and distribute workload.
- Provide suitable time allocation.
- Plan how patients can be involved safely and appropriately.
- Fit into existing medication safety practices, building on other prescribing work and learning across the team.

A number of the PSIs in our intervention involved medicines that are commonly associated with dependence or withdrawal. Medicines such as these need to have careful management to minimise dependency and manage withdrawal.^39 40^ This intervention may, therefore, be useful in providing more enhanced monitoring of patients prescribed these medicines.^41 42^

### Strengths and limitations

Our findings relate specifically to prisons within England. We acknowledge that any translation of these findings to other prison settings would need further research. A particular strength of this study is the utilisation of NPT to understand how the intervention was implemented. Our approach was to see the connections and links between the data and the components of NPT and thus try to understand the ways in which the intervention was being brought into everyday practice. Undertaking this study in prison settings while there were still restrictions associated with the COVID-19 pandemic and impacts of it at local prison level was one challenge. Prison staff did report that workload was difficult, that staffing levels had been impacted by the pandemic and that resources, including staff time, were stretched. This could have, therefore, adversely affected our recruitment figures for the interviews and the survey, as well as responses to survey questions where only free text data from the survey was useful, since take-up of the survey was low and it was only partially completed by some participants. In some prison sites, the intervention had not been rolled out because of staffing issues, and in others, there were significant workload issues that impacted on progress implementing the intervention and willingness and availability to take part in the evaluation. These are important considerations when undertaking evaluations of healthcare interventions within prison settings.

## Conclusions

This qualitative study using NPT enabled us to understand the implementation of an intervention for medication safety in prison settings. As an intervention package the suite of PSIs and the work of MDTs to undertake medication safety actions in response to the reports they generated was dependent on individual leadership and collective multidisciplinary working. New work processes and practices were instigated in order to implement the intervention often fitting into existing medication safety practices, building on other prescribing work and creating learning across the team. As such the implementation was a social practice. While we found that prison staff reported challenges to implementation that should be considered in future, similar interventions may be used for prescribing safety in prison settings.

## supplementary material

10.1136/bmjopen-2024-086309 online supplemental file 1

10.1136/bmjopen-2024-086309 online supplemental file 2

10.1136/bmjopen-2024-086309 online supplemental file 3

## Data Availability

All data relevant to the study are included in the article or uploaded as supplementary information.
