# Supplementary material for: Understanding the implementation of a multidisciplinary intervention using a suite of prescribing safety indicators to improve medication safety in prison healthcare settings: a qualitative study
Source: BMJ Open. 2025 Mar 5;15(3):e086309. doi: 10.1136/bmjopen-2024-086309 (PMC11883610; doi:10.1136/bmjopen-2024-086309)
Supplement: online supplemental file 1 [file bmjopen-15-3-s001.docx]

**A multi-disciplinary approach to reducing potentially hazardous prescribing in prison settings: A mixed method evaluation.**

I am [….] and I am a researcher at The University of Manchester. This study involves the use of a new suite of prescribing safety indicators developed specifically for prison settings. These indicators have been employed in prisons run by the Practice Plus Group and form part of a wider improvement intervention which is described as follows. A ‘prescribing safety indicator champion’ from each prison site will run the prescribing safety indicator searches and presents the findings to the multi-disciplinary team during existing ‘Safer Prescribing’ and ‘Multi-Professional Complex Case’ meetings in order to respond to the data. The multi-disciplinary team may then continue to meet and respond to the indicator data over time, whilst the ‘champion’ also runs new indicator searches. You are being invited to take part in semi-structured interviews to explore the implementation, use, impact and sustainability of using and responding to prescribing safety indicators in this way as part of the multi-disciplinary team to reduce the burden of hazardous prescribing in prison settings. We are looking to gather the experiences of a range of stakeholders involved in implementing and reviewing/responding to the prescribing safety indicator data, such as pharmacists, pharmacy technicians, doctors, psychiatrists, nurses, and non-healthcare staff such as security officers or Practice Plus Group SystmOne data analysts. You are being invited to take part in interviews because you may have been involved in the operationalising, implementation or review/responding to prescribing safety indicators as part of your work throughout this study.

This interview will last for up to one hour. I should remind you that the interviews are confidential. I’d like to record through MS Teams the discussion if that is okay with you; this is simply to help me capture all of the information that comes out of it. If you prefer, I can make written notes instead. You can ask for the recording to be switched off at any point during the interview. The recordings will be destroyed as soon as they are transcribed, the transcripts will be kept in a secure location for five years after the study is complete and then they will be destroyed.

Before we begin, I’d like to provide some ground rules for the discussion:

- You are being digitally recorded, so speak clearly;
- We will anonymise the transcript so that nobody can be identified by name. However, please try to avoid naming specific people or locations;
- Everything discussed here is confidential. However, if you were to reveal anything that would be considered unlawful or anything that would place you or somebody else at risk of harm, we may have to report this to a clinical supervisor or manager. Unless you have any questions for me, then we can begin.

Interview Questions

1. What is your role within this organisation?
2. Which prison do you work in?
3. Were you tasked with a specific responsibility in implementing this intervention? What was your role in the intervention?
4. Can you briefly tell me how the intervention was delivered in your organisation?
   1. Who conducted the PSI search?
   2. Who was involved in the intervention from your team?
   3. How often did the team(s) meet to address the PSI search results?
   4. What did they do and how was this done? How did you use the information obtained from the PSI search?
   5. What did you do as a team to implement this intervention?
   6. What generally happened after these meetings?
   7. Were non-healthcare staff involved in responding to the indicator data and if so how?
5. PSI Champion: how did you work on the data received from the analyst and then present this to the wider team? Did you face any problems with receiving/analysing/presenting the data – please describe these
6. What are your reflections on how you / the team responded to the new PSI search results in your prison(s)?
   1. What went well / not so well and why?
   2. Does this process differ to what was done before with regards indicator response, and if so how?
7. What did you/your team understand were the aims of receiving and discussing the indicator data at your prison, and what benefits (if any) did you think this would achieve? How did you/your team reach this understanding?
8. How did you/others find integrating this work into your usual routine and prison structures?
   1. Were any adjustments required / what impact on your work did this have?
   2. Were all the activities carried out as expected?
   3. Would you do anything differently?
9. Were you/the team motivated to continue working on responding to the data over time, and what influenced this?
10. Did the intervention have any impact on the patients? If so, can you give some examples? How did you find patients response to this intervention? What was done to check whether the intervention had an impact on patients/whether the intervention was effective? What is the most important aspect of the intervention which led to achieving this impact (or how can the intervention achieve an impact in the future)?
11. How well do you feel the intervention was implemented in your prison, and what factors influenced this and why? Do you feel this intervention is sustainable and why?

Interviewer conclusion:

Concluding questions

- Is there anything that you would like to talk about?

- Is there anything that you would like to go back and talk about?

*End recording*

Many thanks for taking the time to help us with this study. Your contribution has been extremely valuable. If you wish we can send you a copy of your interview transcript, and you can also request to receive a summary of the findings of this research study, just ask us. In the meantime please feel free to contact either myself or the other researcher(s) involved if you have questions in future.
