# Supplementary material for: Understanding the implementation of a multidisciplinary intervention using a suite of prescribing safety indicators to improve medication safety in prison healthcare settings: a qualitative study
Source: BMJ Open. 2025 Mar 5;15(3):e086309. doi: 10.1136/bmjopen-2024-086309 (PMC11883610; doi:10.1136/bmjopen-2024-086309)
Supplement: online supplemental file 2 [file bmjopen-15-3-s002.docx]

**A multi-disciplinary approach to reducing potentially hazardous prescribing in prison settings: A mixed method evaluation.**

**Survey**

**About this research**

Prescribing safety indicators are statements of potentially hazardous prescribing events that may place patients at risk of harm. The use of prescribing safety indicators is one way to proactively identify potentially hazardous prescribing and to reduce the risk of harm associated with medication in healthcare settings. This study involves the use of a new suite of prescribing safety indicators developed specifically for prison settings. These indicators have been employed in prisons run by the Practice Plus Group and form part of a wider improvement intervention which is described as follows. A ‘prescribing safety indicator champion’ from each prison site will run the prescribing safety indicator searches and presents the findings to the multi-disciplinary team during existing ‘Safer Prescribing’ and ‘Multi-Professional Complex Case’ meetings in order to respond to the data. The multi-disciplinary team may then continue to meet and respond to the indicator data over time, whilst the ‘champion’ also runs new indicator searches.

This survey is designed to help get a better understanding of how to apply and integrate a multidisciplinary approach to reducing potentially hazardous prescribing in prisons using data from this new suite of prescribing safety indicators.

This survey asks questions about the implementation of this new suite of prescribing safety indicators in prison settings, and how the multidisciplinary team works to reduce the prevalence of hazardous prescribing as defined by these indicators in their local prison(s). We understand that people involved in this intervention have different roles, and that people may have more than one role.

**Why are you being invited?**

Your local manager or Chief Pharmacist may have helped us to identify you as someone who could contribute to this study because of your direct involvement in the intervention.

**Your participation in this survey is voluntary**

It is up to you to decide whether or not to take part. If you decide to take part you are still free to withdraw at any time without giving a reason and without detriment to yourself. However, it will not be possible to remove your data from the project once it has been anonymised as we will not be able to identify your specific data. This does not affect your data protection rights. If you decide not to take part you do not need to do anything further.

***Are there any risks or benefits in taking part?***

There are no direct benefits to you from taking part in this study. However, by taking part you may reflect on how the team (e.g. data analysts, healthcare and non-healthcare staff, patients), local processes and features within SystmOne might work better to improve the quality and safety of medication use. The study findings will also help us to understand how similar interventions might be used in a complementary way in order to produce the most positive effect on patient safety. There are no risks anticipated in taking part in this research. However there is a time burden of approximately 20 minutes so it might be useful to plan ahead.

**What information will be collected and how it will be used and stored.**

In order to participate in this research project we will need to collect information that could identify you, called “personal identifiable information”. Specifically we will need to collect:

- The name of the prison you work in
- Your role in that prison
- Your opinions on the intervention

Only the research team will have access to this information. In accordance with data protection law, The University of Manchester is the Data Controller for this project. This means that we are responsible for making sure your personal information is kept secure, confidential and used only in the way you have been told it will be used. Only the researcher and study co-ordinator, Dr Mark Jeffries , will have access to the data collected in the study. Data obtained from this study will be retained for 5 years.

Please also note that individuals from The University of Manchester or regulatory authorities may need to look at the data collected for this study to make sure the project is being carried out as planned. All individuals involved in auditing and monitoring the study will have a strict duty of confidentiality to you as a research participant. All data will be stored securely either electronically on encrypted password protected files on a secure server and/or in locked filing cabinets on university premises that is only accessible by members of the research team.

**Who has reviewed the research project?**

This study has been approved by NHS Research Ethics Committee (date and ref) and Her Majesty’s Prison and Probation Service (date and ref).

**Contact details for complaints**

If you have a complaint that you wish to direct to members of the research team, please contact**: DR MARK JEFFRIES** by email to [mark.jefferies@manchester.ac.uk](mailto:mark.jefferies@manchester.ac.uk)

**If you wish to make a formal complaint to someone independent of the research team or if you are not satisfied with the response you have gained from the researchers in the first instance then please contact**

The Research Ethics Manager, Research Office, Christie Building, The University of Manchester, Oxford Road, Manchester, M13 9PL, by emailing: [research.complaints@manchester.ac.uk](mailto:research.complaints@manchester.ac.uk)  or by telephoning 0161 306 8089.

If you wish to contact us about your data protection rights, please email [dataprotection@manchester.ac.uk](mailto:dataprotection@manchester.ac.uk) or write to The Information Governance Office, Christie Building, The University of Manchester, Oxford Road, M13 9PL at the University and we will guide you through the process of exercising your rights.

You also have a right to complain to the [Information Commissioner’s Office](https://ico.org.uk/concerns) about complaints relating to your personal identifiable information (<https://ico.org.uk/make-a-complaint/>) Tel 0303 123 1113

**How the findings of the study will be made available**

The results of the study will be published in reports to be held at the University of Manchester. It is our expectation that the results will also be published in journal articles, online reports and in conference presentations that will be released into the public domain. No participant will be identified in any publication. If you wish, we can provide you with a copy of the final study report when the work has been concluded.

**Survey Instructions**

The survey will take 20 minutes to complete.

From the statements below, please choose an option that best describes ***your main role*** in relation to the intervention.

- I am the delegated prescribing safety indicator champion involved in conducting, analysing and preparing the prescribing safety indicator report to present to team members
- I am involved in delivering the intervention by responding to prescribing safety indicator results to work towards reducing the number of patients affected by them
- Other (please describe below)

For this survey, please answer all the statements from the perspective of your main role in relation to the intervention that you have identified above. Depending on your role or responsibilities in the intervention, some statements may be more relevant than others.

The survey is in three parts. Part A asks some brief questions about yourself and your role. Part B includes three general questions about the intervention. Part C contains a set of more detailed questions about the multidisciplinary intervention. For each statement in Part C, there is the option to agree or disagree with what is being asked (OPTION A). However, if you feel that the statement is not relevant to you, there are also opportunities to tell us why (OPTION B).

Please take the time to decide which answer **best suits your experience for each statement and tick the appropriate circle.**

**Part A: About yourself**

Please state the name of the prison(s) you routinely work in

What is your job role within this prison(s)? If you job role is different across prisons, please provide details.

How many years have you worked in this prison(s)? (if you work in more than one prison routinely, please indicate the longest time period you have worked in one of these prisons)

Less than one year (<1 year)

1 year or more but less than 2 years (>=1 year but <2 years)

2 years or more but less than 5 years (>=2 years but <5 years)

5 years or more but less than 10 years (>=5 years but <10 years)

10 years or more but less than 15 years (>=10 years but <15 years)

15 years or more (>=15 years)

How many years have you worked in the prison healthcare sector?

1 year or more but less than 2 years (>=1 year but <2 years)

2 years or more but less than 5 years (>=2 years but <5 years)

5 years or more but less than 10 years (>=5 years but <10 years)

10 years or more but less than 15 years (>=10 years but <15 years)

More than 15 years or more (>=15 years)

Were you tasked with a specific responsibility in implementing this intervention?

**Part B: General questions about the intervention**

Do you have any previous experience of using prescribing safety indicators (in any setting) before using the new indicators involved in this intervention?

Yes

No

How long has the intervention, using the prescribing safety indicators, been in place in your prison?

When you review data about the prescribing safety indicators in this prison setting, how familiar does it feel?

Still feels very new Feels completely familiar

0 1 2 3 4 5 6 7 8 9 10

Brief describe how your organisation responded to the prescribing safety indicator data in your prison, which professional group(s) were involved, and how often meetings to respond to prescribing safety indicator data took place.

Briefly comment on whether you feel that the intervention was worthwhile/improved medication safety.

Briefly comment as to how those delivering the intervention were able to sustain its implementation

**Part C: Detailed questions about the intervention**

**For each statement, please select an answer that best suits your experience using Option A. If the statement is not relevant to you, please select an answer from Option B. If you routinely work in more than one prison, where relevant, please complete the survey questions based on the delivery of the intervention in the prison where you spend the most time working. (There is a comments box below to add comments about the implementation of the intervention in other prisons you work in)**

|  | **Option A** | | | | | **Option B** | | |
| --- | --- | --- | --- | --- | --- | --- | --- | --- |
|  | **Strongly agree** | **Agree** | **Neither agree nor disagree** | **Disagree** | **Strongly disagree** | **Not relevant to my role** | **Not relevant at this stage** | **Not relevant to the intervention** |
| **Coherence** |  |  |  |  |  |  |  |  |
| I can see how the intervention differs from our usual way of working on prescribing safety indicators |  |  |  |  |  |  |  |  |
| Staff in this organisation have a shared understanding of the purpose of this intervention |  |  |  |  |  |  |  |  |
| I understand how this intervention affects the nature of my own work |  |  |  |  |  |  |  |  |
| I can see the value of this intervention for my work |  |  |  |  |  |  |  |  |
| **Cognitive Participation** |  |  |  |  |  |  |  |  |
| There are key people who drive this intervention forward and get others involved |  |  |  |  |  |  |  |  |
| I believe that participating in this intervention is a legitimate part of my role |  |  |  |  |  |  |  |  |
| I have worked with colleagues in new ways to use this intervention |  |  |  |  |  |  |  |  |
| I will continue to support this intervention |  |  |  |  |  |  |  |  |
| **Collective Action** |  |  |  |  |  |  |  |  |
| I can easily integrate the work to identify and respond to prescribing safety indicators into my existing duties |  |  |  |  |  |  |  |  |
| This work to identify and respond to prescribing safety indicators in my prison disrupts working relationships |  |  |  |  |  |  |  |  |
| I have confidence in other people’s ability to use prescribing safety indicator data to improve prescribing practices |  |  |  |  |  |  |  |  |
| Work was assigned to those with appropriate skills to analyse and respond to prescribing safety indicator data |  |  |  |  |  |  |  |  |
| Sufficient training was provided to enable staff to implement this intervention |  |  |  |  |  |  |  |  |
| Sufficient resources were available to support this intervention |  |  |  |  |  |  |  |  |
| Responding to prescribing safety indicators as part of the multi-disciplinary team is currently a normal part of our work routine |  |  |  |  |  |  |  |  |
| **Reflexive Monitoring** |  |  |  |  |  |  |  |  |
| The staff agree that this intervention was worthwhile |  |  |  |  |  |  |  |  |
| The staff agree that this intervention has improved medication safety |  |  |  |  |  |  |  |  |
| I value the effects that this intervention has had on patients |  |  |  |  |  |  |  |  |
| The staff agree that responding to prescribing safety indicator data with patients was challenging |  |  |  |  |  |  |  |  |
| This intervention requires further adaptation to be sustained long-term |  |  |  |  |  |  |  |  |

**Further comments if you work in more than one prison.**

**Please add brief comments below about the implementation of the intervention in other prisons in which you may work**
