# Supplementary material for: Understanding the implementation of a multidisciplinary intervention using a suite of prescribing safety indicators to improve medication safety in prison healthcare settings: a qualitative study
Source: BMJ Open. 2025 Mar 5;15(3):e086309. doi: 10.1136/bmjopen-2024-086309 (PMC11883610; doi:10.1136/bmjopen-2024-086309)
Supplement: online supplemental file 3 [file bmjopen-15-3-s003.docx]

***Supplementary S3.***

***Detailed Results -Themes, Sub themes and Extracts mapped onto Normalisation Process Theory Constructs and Components***

| **Detailed Themes and Extracts mapped onto Normalisation Process Theory Constructs and Components** | | |
| --- | --- | --- |
| **THEME ONE - BRINGING PEOPLE TOGETHER AND ESTABLISHING INDIVIDUAL AND COLLECTIVE ROLES THAT FACILITATED IMPLEMENTATION OF THE INTERVENTION** | | |
| **SUB-THEME 1A - GROUP PARTICIPATION, BUY IN AND WILLINGNESS.** | | |
| **NPT Construct** | **Corresponding Components** | **Interview extracts and Survey free text responses – (Interviews unless stated)** |
| **Coherence**  Sense-making work: understanding and conceptualisation of interventions and their work. | **Communal Specification**  People working together to build a shared understanding of the aims, objectives, and expected benefits of a set of practice. How a team works out how to integrate an innovation into their healthcare setting.  *Do all those involved agree about the purpose of the intervention?* | Monthly meetings with and joined with the local medicines management meetings. I have started working directly with mental health support worker and prescriber, GP and Clinical Lead/Nurse practitioner with specific PSIs ***Lead Pharmacy Technician (Survey free text)***  R: I think meeting regularly as a team of prescribers and professionals has gone really well. I think having our GP, we’ve had such buy-in from the GP, I think that’s been brilliant for us. That’s really helped. I think had the GP not have been so positive, then this would have been far more complicated. So the buy-in from the whole (team) really has been great and then I really think having the senior pharmacy tech almost gatekeeping our prescriptions has been great. That’s been, and that continues now. So I think that’s been the most positive, so regular meetings, buy-in from the team and the gatekeeping from pharmacy, they would be our three main positive aspects. ***Clinical lead***  I suppose it obviously required the willingness of the entire team to be involved, ‘cause with doing it through MPCCC, there’s typically a primary healthcare representation, the inpatient wing, mental health, the recovery team, sometimes pharmacy, GPs, ANPs. So everyone’s kind of got to sit through the MPCCC repeatedly [when there are 00:19:28] lots of opioids and medication issues, when it’s maybe not always relevant to their services, but it’s there for a group discussion. So it requires that group participation and willingness. ***ANP3***    The problem for us, comes with staffing. So here, we haven’t had a pharmacist, there are no other GPs, there are no nurse prescribers, well sorry, we’ve got one nurse prescriber but for, kind of, does a mental health and substance misuse, of which substance misuse is quite small in our prison, and a nursing team whom we have lost several members of, and who are struggling post-COVID. […] So, we didn’t have, so we have a Medicines Management Meeting, which is the formal kind of medicines management operational type meeting, but we didn’t have in place a patient safety regular weekly, monthly, fortnightly, anything meeting. We tried to tag it into…so I presented the whole project at the end of one of the MPCCCs, ‘cause that’s when we have most of the team together, I showed them the reports, I shared the guidance, but after that point we’ve struggled. ***GP*** |
| **SUB-THEME 1AB - WORKING TOGETHER AS A MULTI-DISCIPLINARY TEAM, AGREEMENT OVER ACTIONS AND JOINT RESPONSIBILITY** | | |
| **NPT Construct** | **Corresponding Components** | **Interview extracts and Survey free text responses – (Interviews unless stated)** |
| **Cognitive Participation**  Relational work that people do to build and sustain a community of practice around a new technology or complex intervention: notions of legitimation and buy-in, both in terms of the individuals involved and involving others. | **Initiation**  The work people do to drive forward the new or modified practice. Setting things up and working with others to make things happen.  *Are they willing and able to engage others in the implementation?* | In medicines management meetings we would highlight the number of incidents and trends. I would also share the full information with the lead GP (to identify each individual prescriber) after the meeting, so that these issues could be discussed in managerial/clinical supervisions with the individual prescribers. managerial supervisions with the pharmacist was necessary if an intervention was missed, in order to try to improve practice and ensure understanding ***Patient safety and clinical quality lead (Survey free text)***  I have not been invited to any meetings. The start of this project was last minute and not well communicated in terms of my role. Have had no insight from the MH Leads re any MDT approach on site to support them in delivering this intervention. The Leads have explained that they were handed this as an extra task re data and performance with no real consultation which was disappointing ***Regional MH Lead (Survey free text)***    And then how that’s worked, we’ve done this through a series of…so I took a member of each team, so from the pharmacy team, the GP team, one of the primary care nurses and all the prescribers within (name of prison) and we’ve met monthly to start with, going through the checklist and the action plans. And then we’ve used it alongside the MPCCC so the Multi Professional Complex Case…***Clinical Lead***  Yeah, because when I worked here last time, and I was the only ANP here last time, and there were two or three sessional GPs, in fact there were, no, there were four, maybe four or five that just came in ad hoc and things like that. And all the practice was very silo […] I think, because they worked on separate days, as sessional GPs [...] there was no discussion. […] that’s why we have MPCCC on a Monday, so we have that consistency of approach. We communicate what everyone else is doing, and everyone else is on board with it, as opposed to someone doing something on a Monday, and another doctor doing something on a Tuesday, and never speaking to each other ***ANP3***  So who we’re referring to MPCCC, so we’ve changed that as well, so we’ve identified patients that are prescribed Pregabalin and opioids, co-prescribed as high risk patients, so any prescribing around them needs to be referred to MPCCC, so we’ve got an MDT approach, and it’s not just on one person who takes that kind of responsibility, professional responsibility. So it’s all of us and we all have like…we all have an input into whether the prescription needs to continue or whether it can be safely stopped.  ***Pharmacist***  So within the team we certainly have (name), our pharmacist, clinical pharmacist, also we have our lead GP, Dr (name), and then also…so this was at the more formal meetings, we also have head of healthcare. And we also have representatives from the pharmacy techs as well. But sometimes if we’re talking about a prescription for somebody, then we’d also query, take it to the wider team at handover, so there’s that aspect of team as well […] And I think that works quite nicely at trying to again get everyone’s input and what is safe, because typically when you’re in a consultation room as an ANP or a GP, you only see or hear what you’re getting from the patient, aren’t you. Often if we discuss it at MDT or especially as wider team, then we get more intelligence from what’s happening on the wings, so that’s why often sometimes before you prescribe, we will throw it out to the wider team. ***ANP5*** |
| **SUB-THEME 1C –INDIVIDUAL, OR GROUP, RESPONSIBILITY AND LEADERSHIP FOR TAKING ON KEY ROLE IN IMPLEMENTATION.** | | |
| **NPT Construct** | **Corresponding Components** | **Interview extracts and Survey free text responses – (Interviews unless stated)** |
| **Coherence**  Sense-making work: understanding and conceptualisation of interventions and their work. | **Individual specification.**  Individuals’ understanding of their specific tasks and responsibilities around a set of practices.  *Do individuals understand what tasks the intervention requires of them?* | Pharmacist oversees PSI otherwise no-one seems to be aware about it or what it involves. ***Pharmacy Technician (Survey free text)***  ….when this first started being just out, there was only myself really that was a qualified prescriber. So (name), who is my counterpart, had only just started prescribing, so I didn't think it was fair to him to take on extra responsibilities for looking at other people's prescribing. So it just ended up being myself really that got shoved forward to the front. So I did it by default, really ***ANP1***  …..so yeah, I’m flat out trying to keep things going, and in those recurrent conversations of, how close to burnout am I, so when you add a project that, when I looked at it, all I could see was, I need to run a report, and then I need to look at these patients, and I need to decide whether or not I should prescribe differently, and then I need to review the patient as an addition, potentially, and I need to change the prescribing. I couldn’t see anybody within the team that we have now, that I could really pass much of that workload onto. […] Because with us, particularly, it’s not the methadone ones and the…we had a handful of pregabbers, but those weren’t the high ones it was more, I can’t even remember the indicators now, the PPIs, and naproxens and sertraline combinations, it’s the physical health ones.***GP*** |
| **Cognitive Participation**  Relational work that people do to build and sustain a community of practice around a new technology or complex intervention: notions of legitimation and buy-in, both in terms of the individuals involved and involving others. | **Enrolment**  How participants organise and reorganise themselves and others in order to collectively contribute to the work involved in new practices. This is complex work that may involve rethinking individual and group relationships between people and things.  *Do the stakeholders believe they are the correct people to drive forward the implementation?* | So we’ve got a Safer Prescribing Lead called (name), and she manages the dashboard for the Safer Prescribing. She’s generated that and made it into an Excel spreadsheet of all the prisoners that we’ve got on medication, that she updates intermittently and things like that. But again, her decisions, due to the complexity, and obviously the long term use of some of these medicines, the decisions have often gone through the MPCCC format, with (name) presenting patients, or any other member of the team bringing patients to it ***ANP3***  R: Yeah, so initially it was…so the pharmacy manager, (name), had a keen interest in safer prescribing, but she’s now left, and one of the …me and her had a chat, and one of the things she actually mentioned is it’s very clinical, so it requires a lot of using your clinical knowledge, identifying high risk patients, and she felt that it was more appropriate for the pharmacist to take over. And once I’ve actually looked into it myself and broken down, I’ve had a chance to review the PSI indicators, I do agree with her, I think it’s more best-placed with somebody that’s in a clinical job role. ***Pharmacist***    So I did the training online which was very good that you uploaded onto ESS for us. And once I had a good idea of what it was we were doing, I spoke with our pharmacist, (name), who’s aware obviously (of) PSIs, and I told her what the proposal was, what we would try to achieve. And so I think it took about a week or so going through the searches, trying to figure out how we were doing it, and then…and once I’d got the searches working, then it was just having a little bit of a practice to begin with. And then I think from what I remember, I’d already had enough examples for me to take to the Safer Prescribing Committee, yeah, because this was – when was this – so we had the Safer Prescribing Committee, and at that point I think I just started…I hadn’t completed a thorough search at that point, I don’t remember, I can’t remember. But I presented enough information for everyone on the safer prescribing team to understand what it was we were trying to achieve. ***ANP6*** |
| **Cognitive Participation**  Relational work that people do to build and sustain a community of practice around a new technology or complex intervention: notions of legitimation and buy-in, both in terms of the individuals involved and involving others. | **Legitimation** The work ensuring that other participants believe it is right for them to be involved, and that they can make a valid contribution to it.  *Do they think it is appropriate for them to be involved in the intervention?* | The prison has been without a lead Pharmacist for a prolonged period. This role is now filled within the last 3 months but the position is also being used as a full time Pharmacist. Capacity to fully embed and monitor is limited. The monthly meds management meeting is attended by Dep HOH, prescribers, pharmacy manager and Pharmacist. This should encompass that. ***PSI Head of Healthcare (Survey free text)***  We have no fulltime pharmacist, which is also another slight problem, so we’ve had pharmacy technicians. So, every time I've tried to bring it up at medicine management meetings, or safer prescribing meetings, obviously, for the limited staff we have, we find that most of them normally get cancelled, if I'm completely honest. So, it's a case of me having to nab a GP, if I'm not sure about something, as and when I can. If not, it's normally going off and doing it myself with advice and guidance from head of healthcare, and stuff like that. So, me more saying, this is what I'm going to be doing, have you got any major concerns. Until recently, neither of our head or deputy head of healthcare have been clinical either. ***ANP5***  Yeah, I mean, I could see how it would fit beautifully in, if you had plenty of pharmacy time. So, if you had a pharmacist, some of those would fit beautifully, for them to look at the report, review the notes, see if there’s an obvious reason that the prescribing was as it was. If there’s not, they could get into a pharmacist clinic for a meds review, have that discussion with the patient, use the information provided, and then, if no change is needed, you’re done and dusted, if there’s a prescribing change, it’s a simple task for me going, done the review, this is the information, please script for a PPI or whatever it was, stop this […] And then I’ve just got to go, oh, okay, done. But it’s all, with some of the notes, the amount of time it can take to find the information you’re looking for, historically, for some of the physical health things, is quite a lot, ‘cause they’re hidden in, so why was this person ever started on an antidepressant or an anti-inflammatory, or why haven’t they been put on this, that or the other? It takes some digging sometimes. ***GP***  I: So, as I understand, you were tasked to take on the role of PSI Champion, weren’t you, but there were some…I think you came into it after other people had been doing it. Could you just talk me through how that happened?  R: Yeah, so initially it was…so the pharmacy manager, (name), had a keen interest in safer prescribing, but she’s now left, and one of the …me and her had a chat, and one of the things she actually mentioned is it’s very clinical, so it requires a lot of using your clinical knowledge, identifying high risk patients, and she felt that it was more appropriate for the pharmacist to take over. And once I’ve actually looked into it myself and broken down, I’ve had a chance to review the PSI indicators, I do agree with her, I think it’s more best-placed with somebody that’s in a clinical job role. ***Pharmacist*** |
| **Collective Action**  Operational work that people do to enact a set of practices: organisational resources, training, division of labour, confidence and expertise as well as the workability of the intervention in clinical interaction | **Interactional workability**  The interactional work that people do with each other, with artefacts, and with other elements of a set of practices, when they seek to operationalize them in everyday settings. The impact the new practice has on interactions with each other and/or service users.  *Does the intervention make it easier or harder to complete tasks?* | in medicines management meetings we would highlight the number of incidents and trends. I would also share the full information with the lead GP (to identify each individual prescriber) after the meeting, so that these issues could be discussed in managerial/clinical supervisions with the individual prescribers. managerial supervisions with the pharmacist was necessary if an intervention was missed, in order to try to improve practice and ensure understanding ***Patient Safety & Clinical Quality Lead (Survey free text)***  As part of the weekly checks, pharmacy tech completes it and tasks to prescribers ***Clinical Lead for Substance Misuse/SMS prescriber (Survey free text)***  So the way we actually did it was we made a spreadsheet that, I think has been shared with all the other people as well now, and literally what I do is we look at, so I've got a spreadsheet for each one of the PSIs, so every time I run a report, I can just add people on that are missing off that report. And when we actually looked, lots of them, because obviously it looks in the last three months, most of the ones that have actually, that are coming up on the report aren’t even our prescriptions and they stopped before they even arrived with us. ***ANP1*** |
| **THEME TWO: DEVELOPING NEW TASKS, WORK PROCESSES AND PRACTICES TO MAKE THE INTERVENTION WORK IN EVERYDAY PRACTICE.** | | |
| **SUB-THEME 2A - FINDING SOLUTIONS AND PUTTING NEW SYSTEMS IN PLACE TO INTEGRATE THE PSI INDICATORS INTO WORK PRACTICES** | | |
| **NPT Construct** | **Corresponding Components** | **Interview extracts and Survey free text responses – (Interviews unless stated)** |
| **Cognitive Participation**  Relational work that people do to build and sustain a community of practice around a new technology or complex intervention: notions of legitimation and buy-in, both in terms of the individuals involved and involving others. | **Activation**  The work of keeping the new practices in view and connecting them with the people who need to be doing them. Collectively defining the actions and procedures needed to sustain a practice and to stay involved.  *Can stakeholders identify what tasks and activities are required to sustain the intervention?* | No, it’s been relatively easy actually, because I’ve got it on my system on Favourites, so every month I will refresh it, and I keep a record …because what I do is I’ll jot down what it was last month, so say, for example, last month I did it on 20th, I’ll jot it down and I’ll keep a track of whether our figures are going up or down. So one of the things we’ve introduced as part of the PSI is one of my SMS nurses has just passed her prescribing, so we’re going to be targeting these patients in a safer prescribing clinic which she’s going to be leading on. And one of the reports that she’s got access to is the PSI report that she receives monthly that I send to her, and so she’ll go through the report and then she’ll book these patients into the clinic. And that’s something that we haven’t done, and I think the report’s identified what patients we need to target. ***Pharmacist***  So I think for the last one I started a search and then I put all the patients onto a spreadsheet or Word document, and then I started just working through it, and everything linking back to that date, so I wouldn’t repeat the…I learned early on that if I repeat the search, then it gives you a slightly skewed…it was just good to use those results from that one-off really. And even though it could take a couple of weeks, I just felt it was better to do that than to repeat the search, because some patients might have gone, the maybe new ones. And so I’d just be trying to get through it within a couple of weeks, but lately, I mean, I’ve been on leave, so it took a little bit longer. ***ANP5*** |
| **SUB-THEME 2B – TIME ALLOCATION** | | |
| **NPT Construct** | **Corresponding Components** | **Interview extracts and Survey free text responses – (Interviews unless stated)** |
| **Collective Action**  Operational work that people do to enact a set of practices: organisational resources, training, division of labour, confidence and expertise as well as the workability of the intervention in clinical interaction | **Interactional workability**  The interactional work that people do with each other, with artefacts, and with other elements of a set of practices, when they seek to operationalize them in everyday settings. The impact the new practice has on interactions with each other and/or service users.  *Does the intervention make it easier or harder to complete tasks?* | Like I say I work from home on a Friday for an hour and a half, that’s my hours, so that’s when I do the report so it only takes me about an hour and a half to do the reports and action anything and things like that. So it's not, I think like I say once you’ve actually got on top of it it's not a huge task. It's that initial getting everything in place and then making sure that you run the report. And (name) knows how to run the report so if for any reason I’m off (name) will be able to run the report for me and ensure that it's all updated. So the actual spreadsheet is on our, [inaudible] so we’ve got a practice team so (name) knows how to run reports and knows how to document them as well. So although I do it it's just part of my role because that’s what I do on a Friday, that’s my job for a Friday. If for some reason I wasn’t here, (name) would be able to still complete and run the reports. So although it's my responsibility, (name) would be able to take it off me if I needed him to. ***ANP1***  Monday, Tuesday and a Thursday I’ll have clinics in the morning, [inaudible 06:57], and we also have a lot of task things coming though of prescriptions, requests for certain things, so we have to be quite flexible. And sometimes…so even if I set an hour aside to do something, there might be an urgent patient to see, urgent tasks, and on Mondays I also have to go to segregation unit which is where we keep prisoners who are basically breaking rules within the prison, just smaller. And then I’m also quite involved in the evenings with new receptions coming in, new prisoners coming, so from about five o’clock I’m actually assessing these new prisoners and their health needs and if they need substance misuse prescribing. So it’s a fairly fluid day and it’s similar on Tuesdays and Thursdays. So I did find, especially with the last few, trying to fit this in, because the moment you lose a bit of continuity, it’s harder to get back into it. ***ANP5*** |
|  | **Contextual integration**  The resource work - managing a set of practices through the allocation of different kinds of resources and the execution of protocols, policies and procedures. Fit between the new practice and overall organisational context, including organisational goals, morale, leadership and distribution of resources (e.g. funding, policy, priorities).  *Do local and national resources and policies support the implementation?* | Meetings are planned to happen monthly. I have arranged multiple professionals to be involved in assisting with these reports, Mental health lead, Mental health support worker, and GP. I am still building on this as I come to work on a new PSI. These have taken some time to review and assess for the patient's due to staffing and clinics/,meetings being cancelled. ***Pharmacy Technician*** ***(Survey free text)***  And that really comes down to, I’m struggling with the workload here anyway, so trying to add another thing on, I just couldn’t do, and without any other prescribers or a pharmacist, or anyone else on board as such, it was hard to try and engage the non-prescribers to see that there was anything that they could do as part of the project. ***GP*** |
| **SUB-THEME 2C - FITTING NEW PRACTICES AND PROCESSES WITH AND ALONGSIDE EXISTING MEDICINE SAFETY PRACTICES AND WORK** | | |
| **NPT Construct** | **Corresponding Components** | **Interview extracts and Survey free text responses – (Interviews unless stated)** |
| **Collective Action**  Operational work that people do to enact a set of practices: organisational resources, training, division of labour, confidence and expertise as well as the workability of the intervention in clinical interaction | **Relational integration**  The knowledge work that people do to build accountability and maintain confidence in a set of practices and in each other as they use them. The impact the innovation has on relationships between different groups of professionals e.g. trust, accountability and responsibility.  *Do those involved in the intervention have confidence in the new way of working?* | So some of the trends that I’ve seen onsite is we’re more focused on safer prescribing. So one of the things before, I mean, I’ve only been here for about two years, and some of the improvements I’ve seen is along the lines of…is polypharmacy an issue, and that was an issue when I first started, we were co-prescribing opioids with patients who were on Methadone. So one of the things we’ve done is actually looked at it, looked at the reports and said…because initially, so what we receive is we receive a dashboard, and our dashboard will say, you’ve got 70 patients on Amitriptyline or you’ve got 120 patients on Methadone and 30 on Pregabalin, but it never gives you a real breakdown, because what these PSI reports do, it gives us a breakdown of the patients that we need to be making…that need to be having these reviews. And so when we’ve gone into these patients, we’ve realised, oh, they’ve not had their six week review, or they’ve been initiated on an opioid and not had their four week review. So that’s one of the massive improvements we’ve seen. And some of the patient feedback we’ve had as well is some of them have said to us, oh, it’s like, you know, you’re checking in more regularly to see if the opioids are working or whether they’ve built a tolerance and they need to come off it. ***Pharmacist***    Well, for me, because of how we’ve used the data from the PSI, I…and I’ve done it in such a way where it doesn’t create massive amounts of workload so we’ve incorporated into meetings that already exist. So I think it’s quite sustainable, I don’t think it’s going to…because I know from speaking to other pharmacists, they’re regularly reviewing them and using the data to focus some of their meetings. So I can see from the site level I will be, kind of, using them, yeah, for a lot of my meetings, so… ***Pharmacist*** |
| **SUB-THEME 2D – DIVISION OF LABOUR TASKS ASSIGNMENT** | | |
| **NPT Construct** | **Corresponding Components** | **Interview extracts and Survey free text responses – (Interviews unless stated)** |
|  | **Skill set workability**  The allocation work that underpins the division of labour that is built up around a set of practices as they are operationalized in the real world. Who gets to do/did what, and how the tasks relate to their existing skill sets.  *Do those implementing the intervention have the correct skills and training for the job?* | So with regards to the mental health ones, we've been speaking about those because of the compliance with them not having the bloods and everything done. The psychiatrists are well aware. And then we do highlight it to the mental health team that we've asked for the bloods to be taken and they've refused, so there is information that's shared following the PSI where we've asked for the bloods to be done and they’re not being taken. And the mental health team, and we've got a pharmacy tech up there called (name), who’s absolutely brilliant, so again, she’s now embraced it. So it was one of our biggest lists, the mental health and the bloods, so rather than me have to do it, she knows her patients better than what I do, so she did the original scan of all the notes to make sure they had the bloods, lipids and weights taken. ***ANP1***  So we noticed a trend in a lot of our prescribing was we were getting a lot of requests for mirtazapine and actually our mirtazapine prescriptions, although they’re not massively high, our numbers aren’t huge here, but they were bigger than we wanted them to be. So we asked mental health to get involved to stop, it almost felt like we were getting referrals to the GP for mirtazapine and they’d maybe seen a mental health colleague who’d suggested see the GP to start you on mirtazapine rather than say see the GP to talk about your options as the first line of treatment. So we were able to speak to our colleagues to stop that from happening and then the GP said she really wanted to look at her mirtazapine prescribing on the back of the safety indicators. She didn’t feel that this was first line and that it wasn’t always the best or safest option. And also then on the back of that, we got some intel to suggest that there was some diverting going on. Obviously it’s very dry here at the moment because of COVID so people were trying to conceal their mirtazapine, so it all worked full circle, really. ***Clinical Lead***  And we were trying to drive that forwards, so that’s kind of my understanding of it, is that the nursing team are the central part, and the GP is there more for advice, referral to, seeing the complex, and holding that more kind of consultancy role, it was described to me as. But that takes a lot of work to move some of the Band 5s and 6s, who haven’t maybe had any specific training in history taking, examination, coming up with a possible diagnosis, to move towards, and then when COVID hit, that all stopped, ‘cause everyone was running round like headless chickens, and to try and get back to anywhere near that point is really difficult. So I think if we were running to the vision of true nurse-led service, and had engagement with a nursing team felt that the patients were their patients […] that may change how they view getting involved in prescribing issues. ***GP*** |
| **THEME THREE:** **SEEING THE BENEFITS AND VALUE OF THE INTERVENTION AND NEW WORK PROCESSES WITHIN THE CONTEXT OF PRISON HEALTHCARE PROVISION** | | |
| **SUB-THEME 3A – IMPACT UPON COMMUNITIES OF PRACTICE BY CONTRIBUTING TO WIDER MEDICATION SAFETY WORK AND INDIVIDUAL AND GROUP LEARNING.** | | |
| **NPT Construct** | **Corresponding Components** | **Interview extracts and Survey free text responses – (Interviews unless stated)** |
| **Coherence** | **Internalization -** Work to understand the value, benefits and importance of a set of practices. The *w*ork people do to attribute worth to a new way of working.  *Do all the stakeholders grasp the potential benefits and value of the intervention?* | Yes it has made me notice the prescribing trends more ***Pharmacist (Survey free text)***  So I think with the PSI reporting, especially with bloods and things it makes us more aware and it's something, you know, that we probably wouldn’t have been aware of before. So it's all, we’re able to integrate it into our practice whether we’re prescribing these medications or where you see patients who are new in, haven't had bloods for X number of months. So, it allows us to ensure safety for the patients. And it's not about, stopping meds as to whether they are right or wrong, it is exactly down to that, it's patient safety. So I think more than anything, this has just helped us to be safer prescribers. ***ANP1***    …it's making us more aware of some of the issues that previously might have just gone and been ignored, because it's been repeated month after month. It’s like, well, they were on it last month, so we'll give it to them again this month, kind of attitude. Whereas this has actually made us reconsider some of those patients […] so, for example, picking up on the Pregab who doesn't actually need it, but he's been on it for, what, 12, 15 years, and he came in on it, and no one's actually thought about going, well, why is he on it? It’s just carried on purely because of the fact that he had always been on it. […] But that's given us the prompt to actually do that again, which is good. ***ANP4***  I think it’s kind of what it says on the project isn’t it? […] it just seems quite a good way of picking those things up. If felt quite non-judgemental, I didn’t feel, when I saw the reports, or looked at them, or was like, oh, I’ve got patients there. It didn’t feel like anyone was doing it, to kind of beat me over the head and say, you’ve done bad prescribing on this […] But it didn’t come across ever as that. I think it’s just a good systems check isn’t it? And I think once some of those things have become more embedded, it almost has the sense that you then start going, okay, I just need to do, I’m giving you one of these drugs, I need to add in this, because…and I know the background, ‘cause someone’s showed it to me, I haven’t had to go and find it. Someone’s done the hard work and the evidence, I can just read it and go, okay, this is my spiel now […] And that, in due course, you could almost go, okay, we’ve embedded that one, let’s add a new one in, and kind of slowly, over time, you would build up your kind of prescribing safety and development, ‘cause it would become a bit more natural, I think. ***GP*** |
| **Reflexive Monitoring**  Appraising and monitoring implementation work. The appraisal work that people do to assess and understand the ways that a new set of practices affect them and others around them. | **Systemisation**  The work of collecting information in a variety of ways to determine how effective and useful the new practice is for them and for others.  *Will stakeholders be able to judge the effectiveness of the intervention?* | So I think maybe when other things come in, I don’t know what other plans might well be, looking at, so I know one of the things we were looking at because we know we’re quite good with this is our mirtazapine prescribing. I know that’s not one of your PSIs but that’s our most tradable medication and it's only when we were looking at how good we are with the PSI stuff that we then started to looking at things that we might not be quite so good at. So we’ve actually taken on board looking at our mirtazapine prescribing and we’ve altered the way that we prescribe that now. And I think that’s just down to the fact that we’re so good with the safer prescribing stuff that we are now picking up on things that we might not be quite so good at. ***ANP1***  I guess our communication is better and I think maybe the PSI sort of focused us to look at that better than we were, so it’s not anything ground-breaking, I guess. It’s just that we’re all looking at the same focus and none of us want to work in silo as prescribers and we want to be able to support one another and want to be doing the right thing […] and the MPCCC really helps with that as well because then even colleagues that aren’t at the PSI meetings are often going to be at the MPCCC so they then get to know what’s happening and why ***Clinical Lead***    So in terms of adjustments, I think it’s all been positive really, I’ve got nothing negative to report back on. I don’t think…in terms of workload, I don’t think it’s increased workload massively, I think the reports are easy to run, I mean, they highlight the patients you’re looking to look into, it’s quite straightforward. In terms of changes to our meetings, I think as a team, as a healthcare team we’ve benefited, because it’s not just presenting figures, because that’s something that…some of the meds management meetings, it’s…I found that we were just presenting figures with no background information. Whereas now, I can say, well, this report shows this and I’ve gone into the patients’ record and I’ve found this and I’ve looked into some of the reviews they’ve had and they’ve not had a review for this. ***Pharmacist***  So one of the things it helped identify, and that’s something that I took to my pharmacy meeting and shared some of the learning with my pharmacy team, was it identified patients that were prescribed NSAIDs and then were given NSAIDs as part of the minor ailments protocol. So I was able to do quite a lot of learning with that and say, look, you know, the report’s identified that so-and-so’s prescribed Ibuprofen, but yet you’ve given him Ibuprofen gel on minor ailments, and kind of identified that risk. So that was a lot of learning for…some invaluable learning for my technicians as well, from the report, so yeah. Sorry, I’ll stop talking now because I think I’ve gone for ages. ***Pharmacist*** |
|  |  | the opioid reports and the Pregabs, so that’s something that we’ve moved forward with. ***Pharmacist*** |
| **SUB THEME 3B - THE CHALLENGES OF OPERATIONALISING THE INTERVENTION IN PRISON SETTINGS** | | |
| **NPT Construct** | **Corresponding Components** | **Interview extracts and Survey free text responses – (Interviews unless stated)** |
| **Reflexive Monitoring**  Appraising and monitoring implementation work. The appraisal work that people do to assess and understand the ways that a new set of practices affect them and others around them. | **Communal appraisal**  Participants work together - sometimes in formal collaboratives, sometimes in informal groups to evaluate the worth of a set of practices.  *How will stakeholders collectively judge the effectiveness of the intervention?* | It obviously took a lot of time doing complex letters and obviously, when you write to someone to tell them that you’re reducing their medicine, inevitably it starts a paper war, where they start writing long letters of complaint back to you […] We’ve had a few malicious threats about it, so we’re aware of security issues and safety issues, should I say, so there’s a couple of complex patients that are still challenging some of our decisions through the MPCCC, and we’ve had regional input that we’re going to kind of hold an MDT with the patient and a number of the healthcare team, and a couple of members of the prison team, to make sure that everything’s above board. ***ANP3***  We literally have to go and see them face to face, and obviously they’re high risk offenders in the main, we’re a Cat A prison, and it was COVID, so to see someone face to face is better. And we’ve started doing that more and more, or even just speaking at a cell door, is often the way that I will do it now, where, yeah, there’s maybe a little bit of confidentiality, it’s less ideal, ‘cause you’re on the wings, but it means my safety’s guaranteed, ‘cause I can talk to them. And if it goes well, that’s great, if it doesn’t go well, then I’ll have to make a follow up plan for it and things like that, but yeah, we don’t have in-cell phones. ***ANP3***  Would I do anything differently, so…yeah, so what I’ve…I think for the PSI indicators, it’s made me realise that patient engagement is really important from the very beginning. So that’s something that’s kind of missed really, because I see it as like a missed opportunity because I think with these indicators, you realise that these patients need to be more involved from the very start, so they’re aware of the risks of taking these drugs and they’re not just prescribed and put on a repeat. So that’s something we’ve changed as well, so before a doctor initiates an opioid, one of the things I’ve picked up from the notes is they’re explaining the risks of being on a long-term opioid, and that’s reflecting on their entries in the SystmOne. ***Pharmacist***    So, it's very difficult, in certain situations, to make sure that everyone's going to be safe in a situation when you know it could turn into a volatile one, especially when you're talking about altering Pregab or Gabapentin, or one of their medications where they're reliant on it, sleeping tablets, and sedatives, and things like that. It can be quite a hard thought for them, that they'd have to give something like that up. Not only…even if they're not trading it, it's something that they know, in the back of their mind, well, if I'm really stuck for money, I can sell this. A lot of them won't, majority of the time, because they do get caught if they do, and then they know that I come down on them like a tonne of bricks and they get it stopped. But yes, that's always one of the issues that you have within the prison setting. ***ANP4***  I think a lot of our ladies don’t always feel listened to, and so often if you can…if you bring them in and it’s something they’re not even aware of, it can go so far to just reassure them as healthcare we are monitoring them and we are trying to help them. Although in honesty, if you’re talking to a lot of…some of our ladies, they were so dependent on medication that sometimes you can come up against a bit of a wall, because the first thing they feel like is, right, I’m going to stop your meds. And I think once you get past that and they’re aware it’s just a talk, it’s just to ensure that they feel safe, they feel that they’re on the right dose. ***ANP6***    I’d certainly say so, I mean, a lot of ladies will report very traumatic backgrounds, you know, we’ve got ladies who are sex workers, it’s…and very vulnerable group. And have not had access to healthcare, then they come in, we detox them off alcohol, put them on methadone, if they’ve been on benzos, we detox them, and as I say, that’s when they become aware of all these problems, and a lot of it is because you take away their illicit drugs, you take away their coping mechanisms with life, and again I think they seem to maybe think it’s a tablet that solves that. ***ANP6*** |
| **SUB THEME 3C - THE IMPACT IN LIVES OF PATIENTS** | | |
| **NPT Construct** | **Corresponding Components** | **Interview extracts and Survey free text responses – (Interviews unless stated)** |
| **Reflexive Monitoring**  Appraising and monitoring implementation work. The appraisal work that people do to assess and understand the ways that a new set of practices affect them and others around them. | **Individual appraisal**  Individuals appraising the new practice in relation to their own work; the impact it has on their tasks. Actions through which individuals express their personal relationship with the innovation.  *How will individuals judge the effectiveness of the intervention?* | So terms of impact, I think it’s…so it comes down to patient safety really, it’s making sure that the medication they’re receiving they’re benefiting from and it’s not causing them harm. So, I mean, so, for example, I’ll give you an example, so a patient, we recently did…so Mirtazapine’s an antidepressant, it comes under antipsychotics as well, so one of the reports when we pull…so when we pull the PSO4 report, the nurse came to have a word with me saying that she’s actually gone in to do some weights for somebody and realised his weights per month increased because Mirtazapine, one of the side-effects is weight gain. So she was able to bring him in for a review, have a chat with him, see how he feels about the weight gain, and he actually…one of the outcomes from the review was it was quite distressing for him, the weight gain. So we swapped him onto a different alternative. ***Pharmacist***  Definitely, it’s a massive improvement. You can see it in, not only in our numbers, but you can actually see it in the way that we're looking at the prescribing now. It was very much before a case of, well, we’ll do this, see if this works, but people weren't looking at the whole picture of the patient holistically, that's why we had the patients who were on opiates and Pregab. And I had one, I'd never met the patient before, thankfully, in some respects, he was on Pregab, Gabapentin and Methadone, and you're like, why is he on all of this, what's going on. So, to be able to find those patients and actually improve their treatment care and their polypharmacy, it’s a massive difference. And I think some of these patients actually feel better for it as well now. ***ANP5***  The point of checking these PSIs, well, it’s all about patient safety, isn’t it, it’s about ensuring that patients are receiving effective care, safe care, and it also helps to identify patients who would benefit from a meds review, because I think despite everyone’s best efforts, you would always get a patient who may have a repeat prescription or an acute prescription which has just been continued, and it just needs someone to have a look and say, wait a minute, we need to get this patient in. We do try and do that here, but I think certainly the benefits of having this where you’ve got afforded time to look through flagged patients, I think it’s so worth it, it really is. For some patients who haven’t had a review for some time, by allowing us to bring them in, we can actually address those health needs, and again, as I say, it comes back down to patient safety, doesn’t it? So if they’re on a non-steroidal long-term with an SSRI, we can start them off on gastric protection, but of course the question can be, do you need to be on an SSRI for so long maybe. ***ANP6*** |
| **SUB THEME 3D – STAFF CHALLENGES, ADAPTATION AND NEED FOR RESILIENCE IN THE TEAM** | | |
| **NPT Construct** | **Corresponding Components** | **Interview extracts and Survey free text responses – (Interviews unless stated)** |
| **Reflexive Monitoring**  Appraising and monitoring implementation work. The appraisal work that people do to assess and understand the ways that a new set of practices affect them and others around them. | **Reconfiguration**  The appraisal work by individuals or groups which may lead to attempts to redefine procedures or modify practices - and even to change the shape of the innovation itself.  *Will stakeholders be able to modify the intervention based on evaluation and experience?* | Due to capacity, high volumes of other initiatives, and other work pressures such as Covid management, I am not sure this was fully embedded beyond practices that were already in place. ***Head of Healthcare*** ***(survey free text)***  As far as I am aware, this has become another data task. I do not get the impression that good quality MDT approach is in place and I have witnessed on some sites, prescribers changing medication without any consultation with their MH colleagues ***Regional MH Lead(survey free text)***  a few months ago, we kind of got a little bit fatigued with it ourselves, ‘cause we were dealing with so many complaint letters around it all, that we were just getting, it was discouraging, shall we say? And we were struggling, so we made a decision then to pause aggressively doing it on MPCCC, but then we’ve just picked it back up naturally as things have come back on board, but I know we’ve done a lot of the work already. So it is hard to maintain, when you’ve got a client group that are demanding certain medications and things. So yeah, you’ve kind of got to have that strength within the team, and resilience within the team, to keep going through that I think, so yeah. ***ANP3***  Pretty much. I had hoped there'd be more meetings, and it'd be more of a multidisciplinary team with everyone getting involved. But I think that is just the way that the prisons are working across the board at the moment, that we are very short-staffed, and it is very hard to get a wide range of disciplines in the prison. So, when people go, they…even if they move from one prison to another, it's very hard to replace. And they're very busy situations at the moment, prisons, just generally. We struggle, not just with the nursing staff and medical staff, but officers are a nightmare as well to get hold off, and things like that, purely because there isn't enough of them. But that's an issue across the board, so you just get on with it. ***ANP5***  The problem for us, comes with staffing. So here, we haven’t had a pharmacist, there are no other GPs, there are no nurse prescribers, well sorry, we’ve got one nurse prescriber but for, kind of, does a mental health and substance misuse, of which substance misuse is quite small in our prison, and a nursing team whom we have lost several members of, and who are struggling post-COVID […] So, we didn’t have, so we have a Medicines Management Meeting, which is the formal kind of medicines management operational type meeting, but we didn’t have in place a patient safety regular weekly, monthly, fortnightly, anything meeting. We tried to tag it into…so I presented the whole project at the end of one of the MPCCCs, ‘cause that’s when we have most of the team together, I showed them the reports, I shared the guidance, but after that point we’ve struggled.***GP***    We’ve had pharmacy technicians. So, every time I've tried to bring it up at medicine management meetings, or safer prescribing meetings, obviously, for the limited staff we have, we find that most of them normally get cancelled, if I'm completely honest. So, it's a case of me having to nab a GP, if I'm not sure about something, as and when I can. If not, it's normally going off and doing it myself with advice and guidance from head of healthcare, and stuff like that. So, me more saying, this is what I'm going to be doing, have you got any major concerns. Until recently, neither of our head or deputy head of healthcare have been clinical either. So, that's more of a case of, watch when I put the cat amongst the pigeons, just be warned, this is going to happen, there’s going to be a lot more complaints.  ***ANP5*** |
