# Supplementary material for: Understanding the implementation of a multidisciplinary intervention using a suite of prescribing safety indicators to improve medication safety in prison healthcare settings: a qualitative study
Source: BMJ Open. 2025 Mar 5;15(3):e086309. doi: 10.1136/bmjopen-2024-086309 (PMC11883610; doi:10.1136/bmjopen-2024-086309)
Supplement: Supplementary file 4 [file bmjopen-2024-086309.reviewer_comments.pdf]

PEER REVIEW HISTORY

BMJ Open publishes all reviews undertaken for accepted manuscripts. Reviewers are asked to complete a checklist review form and are provided with free text boxes to elaborate on their assessment. These free text comments are reproduced below.

ARTICLE DETAILS

Title (Provisional)

Understanding the implementation of a multidisciplinary intervention utilising a suite of prescribing safety indicators to improve medication safety in prison healthcare settings : A qualitative study

Authors

Jeffries, Mark; Abuzour, Aseel S. M; Ashcroft, Darren; Avery, Tony; Langridge, Mark; Francis, Gayle; O'Brien, Amber; Millington, Tracy; Keers, Richard Neil

---

VERSION 1 - REVIEW

**Reviewer**	**1**
**Name**	**Divac , Nevena**
**Affiliation**	**University of Belgrade**
**Date**	**06-May-2024**
**COI**	**None.**

Healthcare workers in prison settings face various challenges in everyday practice. The quality of prescribin practices is specific in these institutions due to limited resources, mental health issues among incarcerated persons, overcrowdedness and other factors described in the manuscript. The research in prisons is also frequently limited due to different legal restictions and uncooperativness of the staff and prisoners. Therefore, this manuscript presents a valuable addition to the understanding and implementation of interventions with the aim to improve prescribing and, consequently, healthcare. The process of translation of the measures into the practice is addressed seriously and the challenges of the implementation of interventions are successfully dentified

---

**Reviewer**	**2**
**Name**	**Nicholls, Gary**
**Affiliation**	**UNSW Sydney, Medicine**
**Date**	**04-Jun-2024**

COI **No competing Interests / COI**

Page 9 'Framework: Normalisation process theory'- please review / clarify.

NPT is concerned with explaining the work people do rather (?? rather / rather than?? or leave out??) and focuses upon the social processes...

Reviewer **3**

Name **Wyer, Mary**

Affiliation **Westmead Institute for Medical Research, Sydney Institute for Infectious Diseases**

Date **24-Nov-2024**

COI **None declared**

Thank you for the opportunity to review this manuscript "**Understanding a multi-disciplinary intervention for potentially hazardous prescribing in prison settings: A qualitative study informed by Normalisation Process Theory**"

This study aimed to understand the implementation of a suite of seven prescribing safety indicators and multidisciplinary intervention to improve medication safety in prison healthcare setting.

This paper was clear, and the use of Normalisation Process Theory was well used to explore the intervention results.

I have just a few comments and suggestions:

- Strengths and limitations
  - These are clear and well balanced; however I believe the authors should state that the results inform changes that could be made to the UK prison system and that translation to other prison systems would need to be researched.
- Introduction
  - Some of the claims made are backed up with UK references and this could be made clearer.
- Methods
  - Figure one is very helpful.
  - Table 2 is also very helpful for those new to NPT.
  - Please check pg 10 of 64 (or pg 9 at bottom of page) line 55 – "collective" not "collection". This sentence marks the end of a long passage about NPT and should have a reference.
- Results
  - This was a long section, but I enjoyed reading it.

    - I would like to understand better what 'Likert scale data was "poorly" completed' means. Were the questions completed only partially? Or did you find that participants did not understand the questions? Was the instrument piloted?
    - Pg 18 of 64 (or pg 17 at bottom of page) line 33 - Could you expand on what this sentence means? "Leadership could also come from groups"
- Discussion
    - Pg 26 of 64 (or pg 25 at bottom of page) lines 15-22. Could you explain why it could be 'potentially limiting to medication safety interventions, and those using PSIs, if they were only led by prescribers, pharmacists or GPs'?
- Finally – please check the paper for spelling and grammar. There are several instances where commas would provide the reader a more seamless understanding of the text.

Thank you again for the opportunity to review this manuscript.

VERSION 1 - AUTHOR RESPONSE

**Reviewer: 1**		
Healthcare workers in prison settings face various challenges in everyday practice. The quality of prescribing practices is specific in these institutions due to limited resources, mental health issues among incarcerated persons, over crowdedness and other factors described in the manuscript. The research in prisons is also frequently limited due to different legal restrictions and uncooperativeness of the staff and prisoners. Therefore, this manuscript presents a valuable addition to the understanding and implementation of interventions with the aim to improve prescribing and, consequently, healthcare. The process of translation of the measures into the practice is addressed seriously and the challenges of the implementation of interventions are successfully identified	Thank you for your comments	No changes required
**Reviewer: 2**		
Page 9 'Framework: Normalisation process theory'- please review /	Thank you for this comment. We have amended the sentence, deleting 'rather'.	

clarify.<br>NPT is concerned with explaining the work people do rather (?? rather / rather than?? or leave out??) and focuses upon the social processes...	This now reads: *"NPT is concerned with explaining the work people do and focuses upon the social processes of intervention implementation and how they become part of everyday practice as new behaviours are adopted into routine work."* NPT is abbreviated here in response to a comment from the editor above.	
**Reviewer: 3**		
Strengths and limitations<br>These are clear and well balanced; however I believe the authors should state that the results inform changes that could be made to the UK prison system and that translation to other prison systems would need to be researched.	Thank you for this comment. We agree that the findings relate to UK prison settings and more specifically to England (which has a separate criminal justice system to other devolved parts of the UK). We have added the following to the strengths and limitations within the discussion section.<br>*"Our findings relate specifically to prisons within England. We acknowledge that any translation of these findings to other prison settings would need further research."*<br>We have added it here rather than at the Strength and Limitations bullet points since the journal guidelines ask for those bullet points to relate specifically to the methods.<br><br>In addition under the *Implications for practice, policy and further research* subheading in the discussion we have added UK to the following sentence:<br><br>*"Our study raises a number of implications for policy practice and further research and supports the wider exploration and use of prescribing safety or quality indicators to assess, or as interventions for improvement in UK prison settings."*	Discussion P27<br><br>Discussion P26
Introduction<br>Some of the claims made are backed up with UK references and this could be made clearer.	We agree that the specificity of these claims needs some clarification. We have made changes to the first paragraph of the introduction, excluding the final sentence which already had a reference to a study having taken place in England. This now reads:	

	"Patients residing in prisons have greater mental and physical health needs than the general population.[1 2] In the UK increases in the proportion of prisoners aged over 50 will likely result in more prisoners suffering from age-related chronic diseases and polypharmacy.[3] The prison population are more likely to have a history of substance misuse.[4] In the UK overcrowding in prisons, diversion of medication, and inadequate staff levels present challenges to the quality of care provided.[3 5 6] Patients residing in prisons have been seen to have a high prevalence of inappropriate prescribing rates and inappropriate polypharmacy.[7] In two separate studies of prisons in England, Hassan et al., found that age-adjusted prescribing of psychotropic medicines was up to 6 times higher for female prisoners and 5.5 times higher for male prisoners than in the general population.[8 9] Elevated prescribing rates were found in the prison population in England for the use of anticholinergics in patients aged over 65 (25.8%), prolonged use of hypnotics, (46.3%) and the use of antiplatelets prescribed with nonsteroidal anti-inflammatory drugs without gastric protection (12.5-16.7%).[10]	
Methods<br>Figure one is very helpful.	Thank you for this observation	
Table 2 is also very helpful for those new to NPT.	Thank you.	
Please check pg 10 of 64 (or pg 9 at bottom of page) line 55 – "collective" not "collection". This sentence marks the end of a long passage about NPT and should have a reference.	We have changed 'collection' to collective. We have added references to May and Finch 2009(reference 19) and May 2013 (reference 21) here at the conclusion of the part that discusses the four constructs of NPT at the end of the sentence that ends *'everyday working practices'.*	
Results<br>This was a long section, but I enjoyed reading it.	Thank you.	
I would like to understand better what 'Likert scale data was "poorly" completed' means. Were the questions completed only partially? Or did you find that	The survey was piloted and based upon previous literature. We have added the text below to the data collection section of the methods and cite an additional	Methods

participants did not understand the questions? Was the instrument piloted?	reference (Finch et al BMC Medical Research Methodology 2018).<br><br>*"The online survey was piloted within the project team, including prison and mental health pharmacists, with minor amendments made to improve clarity before launch. The survey was also based on the established and validated NPT survey tool available online."*<br><br>Several participants only partially completed the survey. Take up of the survey was low given that it was offered to all healthcare staff at the 30 participating prison sites. We have added to the strengths and weaknesses section in the discussion to reflect further on these points. The end of this section now reads:<br><br>"*This could have therefore adversely affected our recruitment figures for the interviews and the survey, as well as responses to survey questions where only free text data from the survey was useful, since take up of the survey was low and it was only partially completed by some participants. In some prison sites the intervention had not been rolled out because of staffing issues and in others there were significant workload issues that impacted on progress implementing the intervention and willingness and availability to take part in the evaluation*."	Discussion
Pg 18 of 64 (or pg 17 at bottom of page) line 33 - Could you expand on what this sentence means? "Leadership could also come from groups"	At times participants reported that leadership could be from a team not an individual. However, reflecting on this sentence we feel it does not actually fit with the points made in the rest of the paragraph about individual leadership and isolation of that leader. We have therefore deleted this sentence.	Results P18
Discussion<br>Pg 26 of 64 (or pg 25 at bottom of page) lines 15-22. Could you explain why it could be 'potentially limiting to medication safety interventions, and those using PSIs,	Here and in previous research it has been found that prescribing safety interventions are seen as solely the responsibility of those with specific responsibility around medicines, for instance pharmacists. Having input from other staff we feel would strengthen the	Discussion P26

if they were only led by prescribers, pharmacists or GPs'?	intervention. We have made some changes to this sentence. It now reads: *"It could be potentially limiting to medication safety interventions, and those using PSIs, if they were solely led by, and seen as the responsibility of, prescribers, pharmacists or GPs, as found previously [16] or as described in the present study by those with a clinical background. This is because wider health care staff may not have had significant input in intervention delivery."*	
Finally – please check the paper for spelling and grammar. There are several instances where commas would provide the reader a more seamless understanding of the text.	We have checked through the manuscript and corrected spelling and grammar where needed. We hope this now makes for a more readable text.	

VERSION 2 - REVIEW

Reviewer **3**

Name **Wyer, Mary**

Affiliation **Westmead Institute for Medical Research, Sydney Institute for Infectious Diseases**

Date **03-Feb-2025**

COI

Thank you for the opportunity to review the revised version of this manuscript. The paper reads very well.
